# Reversible Single-Electron-Transfer to Oxygen in a Stable N-Heterocyclic Carbene Palladium(I) Metalloradical

**DOI:** 10.1021/acs.inorgchem.3c02878

**Published:** 2023-11-22

**Authors:** Georgiana Maties, Pilar Gómez-Sal, Cristina G. Yebra, Román Andrés, Ernesto de Jesús

**Affiliations:** Departamento de Química Orgánica y Química Inorgánica, Instituto de Investigación Química “Andrés M. del Río”, Universidad de Alcalá, Campus Universitario, 28805 Alcalá de Henares, Madrid, Spain

## Abstract

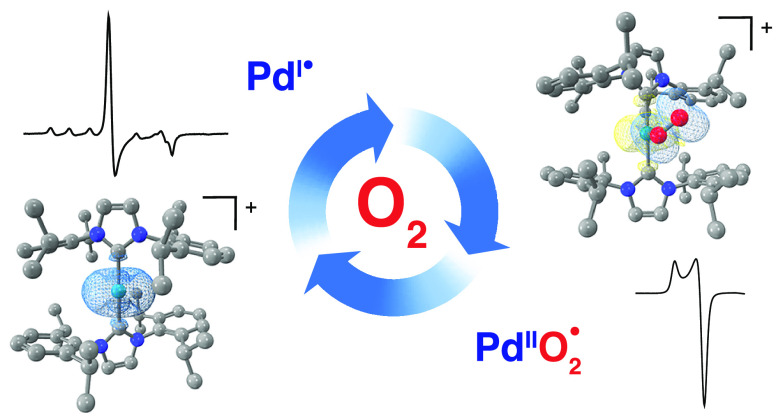

The chemical and electrochemical one-electron oxidation of [Pd(IPr)_2_] (**1**) leads to the formation of mononuclear palladium(I) complex [Pd(IPr)_2_][PF_6_] (**2**). This bench-stable metalloradical has been fully characterized and its structure confirmed by X-ray diffraction analysis. EPR and DFT studies confirm the localization of the unpaired electron onto the metal center. Low temperature NMR and EPR measurements reveal the ability of complex **2** to reversibly coordinate and reduce the dioxygen molecule, leading to the formation of a three-coordinate complex, [Pd^II^(IPr)_2_(η^1^-O_2_)]^+^ (**4**), in which the unpaired electron has been transferred to the superoxido ligand.

## Abstract 2

The synthesis and full characterization of the stable palladium(I) metalloradical [Pd(IPr)_2_][PF_6_] (**2**) is reported. The unpaired electron is located at the metal center according to the experimental (EPR) and theoretical (DFT) evidence. Complex **2** can reversibly bind and transfer an electron to a dioxygen molecule at a low temperature to form the tricoordinate palladium(II) superoxido complex [Pd(IPr)_2_(η^1^-O_2_)]^+^.

Open-shell palladium complexes in oxidation states I and III are receiving increasing attention because they provide alternative methodologies for bond construction through single electron transfer (SET) reactions.^1−4^ Although monometallic palladium(I) metalloradicals are usually transient, Geiger et al. succeeded in 1987 in isolating impure samples of the stable Pd^I^ complex [Pd(η^5^-C_5_Ph_5_)(η^4^-dbcot)].^5^ A new breakthrough came in 2016, when the Chaplin and Ozerov groups characterized independently the stable palladium(I) two-coordinate complex [Pd(P*t*Bu_3_)_2_]^+^ together with its acetonitrile three-coordinate adduct [Pd(P*t*Bu_3_)_2_(NCMe)]^+^.^6,7^ More recently, Deng et al.^8^ and Mirica et al.^9^ reported tricoordinate amido and tetracoordinate dithiapyridinophane palladium(I) complexes, respectively. Beyond these synthetic advances, studies on the reactivity of these stable Pd(I) radicals are still very scarce^10−12^ despite their importance for a better understanding of palladium-catalyzed radical reactions.

We are interested in single-electron-transfer reactions involving stable Pd^I^ radicals as reducing agents for small molecules. In designing suitable complexes to react with dioxygen, we turned our attention to the 1,3-bis(2,6-diisopropylphenyl)imidazol-2-ylidene (IPr) ligand. This bulky N-heterocyclic carbene (NHC) is appropriate to bringing kinetic stabilization to the Pd^I^ centers. In addition, NHC ligands have several benefits over phosphanes for our purposes: (a) NHCs are better donors and could potentially facilitate the oxidation of the metal center from Pd^I^ to Pd^II^;^13−15^ (b) the metal–NHC bonds are far more stable under oxidizing conditions;^16,17^ and (c) two-coordinate palladium(0) [Pd^0^(NHC)_2_] complexes are more widely available^18^ as precursors of the corresponding [Pd^I^(NHC)_2_]^+^ species.

The reduction of dioxygen by [Pd^0^(NHC)_2_] complexes has been previously described as leading to the formation of [Pd^II^](η^1^-O_2_)_2_ superoxides and [Pd^II^](η^2^-O_2_) peroxides.^19−22^ In particular, the reaction of [Pd(IPr)_2_] (**1**) with O_2_ affords the bis(superoxide) *trans*-[Pd(IPr)_2_(η ^1^-O_2_)_2_].^20,21^ Another relevant precedent is the discovery made by Ozerov et al. that photolysis of the Pd^I^–Pd^I^ dimer [Pd^I^(^F^PNP)]_2_ in the presence of an excess of O_2_ leads to the formation of the square-planar superoxido Pd(II) monomer [Pd^II^(^F^PNP)(O_2_)].^23^ Inspired by this work, we considered the feasibility of achieving the formation of palladium(II) superoxide complexes by the single electron oxidation of stable palladium(I) monomers with dioxygen (Chart 1).

**Chart 1:**
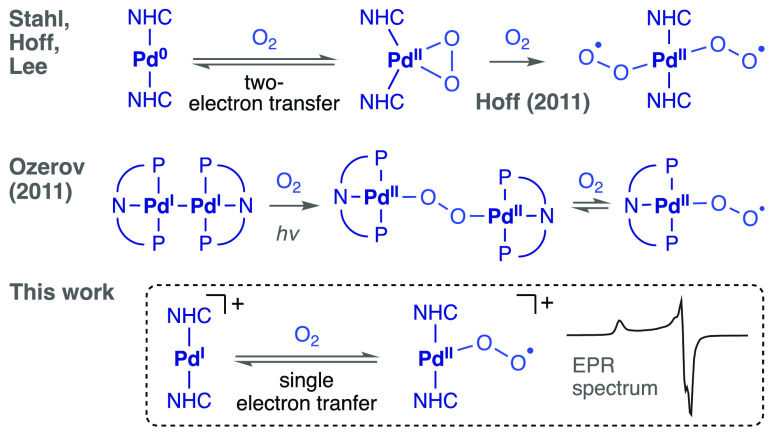


Herein, we communicate the successful isolation and complete characterization of [Pd(IPr)_2_][PF_6_], a bench-stable palladium(I) bis-carbene complex. At low temperatures, this metalloradical can bind oxygen reversibly, leading to the formation of [Pd^II^(IPr)_2_(η^1^-O_2_)][PF_6_], a three-coordinate palladium(II) superoxido complex. This is an unprecedented example of a SET oxidation involving a lone palladium(I) center.

In 1,2-difluorobenzene, [Pd(IPr)_2_] (**1**) undergoes a reversible one-electron oxidation at −1.02 V vs the ferrocenium/ferrocene couple (*i*_p_^forward^/*i*_p_^reverse^ ≈ 1, see Supporting Information).^24^ The chemical oxidation of the Pd^0^ complex with ferrocenium hexafluoridophosphate in the same solvent results in the formation of the stable palladium(I) complex [Pd(IPr)_2_][PF_6_] (**2**, Scheme 1) that was isolated as a pale yellow solid.

**Scheme 1:**
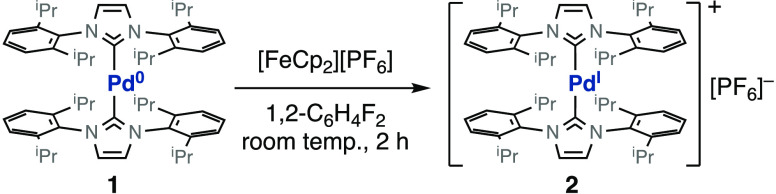
Preparation of the Stable Palladium(I) Radical **2**

The electrochemical potential required to reduce isolated complex **2** is virtually the same as that observed for the oxidation of **1** (−0.99 V vs −1.02 V). This value is approximately 0.55 V less than the one recorded for the palladium(I)/palladium(0) couple in [Pd(P^t^Bu_3_)_2_] (−0.44 V) measured under identical conditions.^6,7^ Therefore, the higher donicity of the carbene ligand significantly facilitates the oxidation of palladium(0) to palladium(I).

The monometallic complex **2** was first identified by the observation of an intense peak in the electrospray ionization mass spectrum corresponding to the [Pd(IPr)_2_]^+^ ion (Figure S3). In addition, the effective magnetic moment (μ_eff_) of 1.68 μ_B_ measured at room temperature by the Evans method in a CD_2_Cl_2_/cyclohexane solution is close to the expected spin-only value of 1.73 μ_B_ for an *S* = ^1^/_2_ spin system. Finally, the molecular structure of **2** was confirmed through X-ray diffraction analysis of yellow crystals obtained from a concentrated solution of the complex in 1,2-difluorobenzene (Figures 1a and S25).

**Figure 1:**
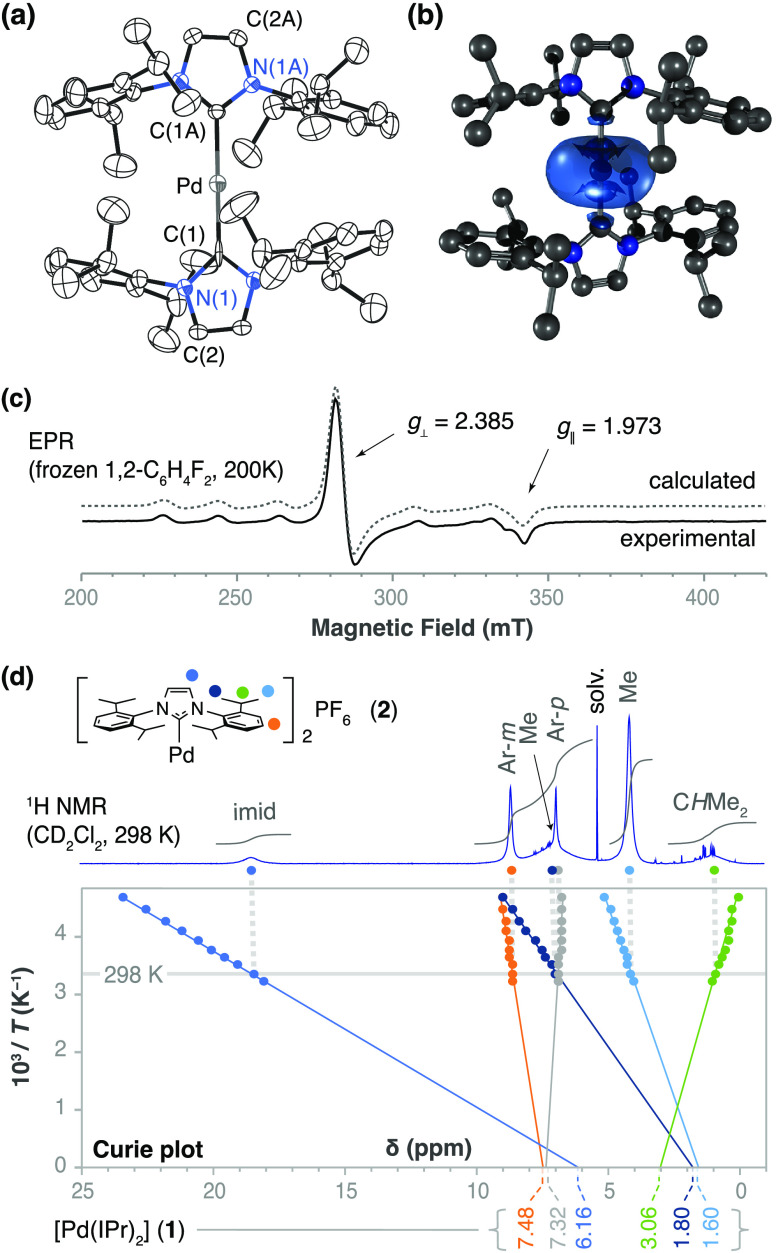
Characterization of complex **2**. (a) ORTEP diagram of the cationic [Pd(IPr)_2_]^+^ unit (50% probability ellipsoids; H atoms omitted). Bond lengths (Å) and angles (deg): Pd–C(1), 2.021(7); Pd–C(1A), 2.091(6); C(1)–Pd–C(1A), 180. (b) Spin density distribution calculated by DFT (B3LYP/LanL2DZ; isosurface plot at 0.001 au; H atoms omitted). (c) Experimental and calculated EPR spectra of a frozen solution in 1,2-difluorobenzene at 200 K.^25,26^ (d) From top to bottom: assignment of resonances in the ^1^H NMR spectrum recorded at 298 K in CD_2_Cl_2_; plot of the chemical shifts as a function of the inverse of temperature (the straight lines represent the best fit of the data to Curie’s Law); chemical shifts for the analogous protons in the diamagnetic Pd^0^ complex **1**.

The structural parameters that define the cationic moiety [Pd(IPr)_2_]^+^ are highly similar to those observed in the neutral Pd^0^ complex [Pd(IPr)_2_] (**1**; see Table S7).^27,28^ The coordination of the NHC rings to palladium is linear (180.0° in **2** and 177.2° in **1**), and their planes form a dihedral angle of 39.4° in **2** and 46.8° in **1**. One exception to this pattern is observed for the palladium-carbene distance, which increases significantly from an average of 2.013 Å in **1** to 2.056 Å in **2**. The linear arrangement of ligands and the elongation of the metal–ligand bond compared to the corresponding M^0^ complexes are common features to other M^I^ two-coordinate d^9^ complexes of group 10 metals.^6,7,29^ The elongation of the metal–ligand distance in the oxidation of [Pt(P*t*Bu_3_)_2_] to [Pt(P*t*Bu_3_)_2_]^+^ was nicely explained by Ozerov et al. in a computational study.^7^

The EPR spectrum, recorded in frozen 1,2-difluorobenzene at 200 K, shows a good match with that of a calculated *S* = 1/2 spin system with axial symmetry and *g*_∥_ and *g*_⊥_ values of 1.973 and 2.385, respectively (Figure 1c). The main resonances are superimposed with the sextuplets arising from hyperfine coupling with palladium-105 (*I* = ^5^/_2_; abundance of 22.33%). The large coupling constant to the metal (*A* ≈ 25 mT) and the high anisotropy of *g* (2.385 versus 1.973) are indicative of a radical mainly located at the metal center. In addition, the relationship *g*_⊥_ > *g*_∥_ corresponds to that predicted for a d^9^ metal configuration with a d_*z*_^2^ SOMO orbital in an axially symmetric structure. The hypothesis was additionally supported by density functional theory (DFT) calculations of spin density, which indicated that the contributions of the ligand atoms to the spin density are negligible compared with those of the palladium center (Figure 1b).

Despite the paramagnetism of **2**, its ^1^H NMR resonances are not very wide (Δν_1/2_ = 41 to 723 Hz) and spread over a relatively narrow spectral window (about 30 ppm in dichloromethane-*d*_2_ at room temperature, Figure 1d). This fact has made easier the complete assignment of resonances based on their line widths, areas, and longitudinal relaxation times *T*_1_. The assignment of the ^1^H resonances depicted in Figure 1 was supported by the variable-temperature NMR data collected between 213 and 308 K (Table S2). Paramagnetic contributions to chemical shifts are temperature-dependent and generally conform to the behavior predicted by Curie’s law.^30^ As a consequence, the chemical shifts of the paramagnetic substances tend to converge to the expected diamagnetic regions at high temperatures. The evolution of the ^1^H chemical shifts of complex **2** with temperature obeys with a good accuracy to Curie’s law, with linear regression coefficients greater than 0.99 in most cases (Figure 1d). The projection of these lines at infinite temperature (1/*T* = 0) is coincident with the regions in which these protons are observed in palladium(0) analogue **1**.

**Figure 2:**
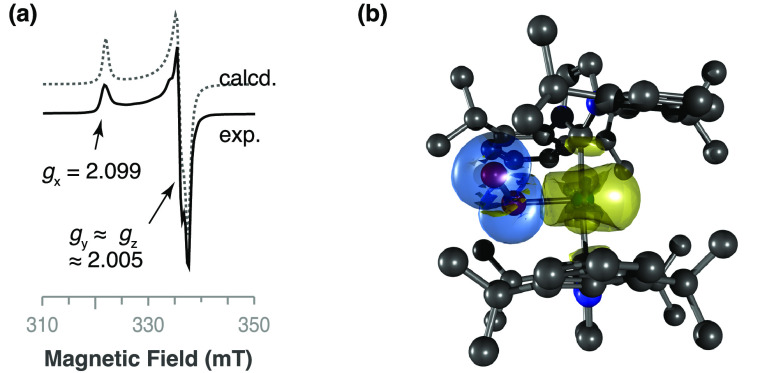
(a) Experimental and calculated EPR spectra of a frozen solution of complex **4** in acetone at 170 K. (b) Spin density distribution calculated by DFT for **4** (B3LYP/LanL2DZ; isosurface plot at 0.001 au; H atoms omitted). The blue surface (positive spin density) and the yellow surface (negative spin density) correspond to an excess of α and β-electron density, respectively.

The ^1^H NMR spectra of complex **2** in potentially coordinating solvents such as acetone-*d*_6_, acetonitrile-*d*_3_, or tetrahydrofuran-*d*_8_ were essentially identical to that described above in dichloromethane-*d*_2_ (Table S1). The EPR spectra of **2** obtained in the solid state and in different frozen solvents at low temperature also exhibit a remarkable similarity (Figure S10). However, the spectrum recorded in frozen acetonitrile shows the partial formation of adduct **3** (eq 1, *g* = 2.328 and 2.087; Figure S10b). Compared to the room temperature formation of the acetonitrile adduct in the case of [Pd(PtBu_3_)_2_]^+^,^7^ the lower temperature at which acetonitrile coordinates to **2** is another indication of the lower acidity and higher electronic richness of the palladium atom in the carbene complex.
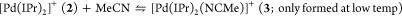
1Complex **2** is stable at room temperature and does not react with air or pure dioxygen in the solid state. In addition, the ^1^H NMR spectrum of the complex in acetone-*d*_6_ remains unchanged upon pressurizing the NMR tube with 1 bar of O_2_ at room temperature (Figure S13). However, the formation of a new paramagnetic complex (**4**, Scheme 2a) is clearly observed in the spectrum upon cooling the solution between 253 and 233 K with **2** and **4** reaching a similar concentration at 213 K. Simultaneously, the yellow color of the solution clearly darkens. The EPR spectrum evidences the complete conversion of **2** into **4** when the solution of acetone is frozen at 170 K. The EPR signals of **4** are characterized by the lack of observable hyperfine coupling to ^105^Pd and by *g* values close to each other and close to the electron *g* factor of 2.0023 (Figure 2a). These spectroscopic data are consistent with the description of **4** as a superoxido complex formed by one-electron oxidation of Pd^I^ to Pd^II^. This view is further supported by DFT calculations of the spin density distribution that show an intense positive spin density on the oxygen atoms together with some negative spin density on palladium (Figure 2b and Table S10).

**Scheme 2:**
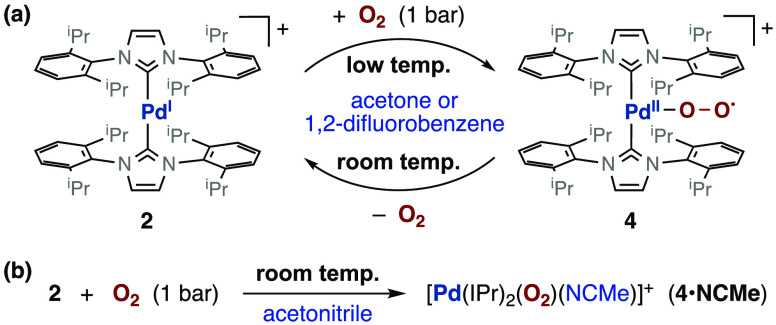
Formation of the Superoxido Complex [Pd(IPr)_2_(η^1^-O_2_)][PF_6_] (**4**)

The binding of dioxygen to the palladium(II) center in **4** is reversible, and complex **2** can be completely recovered by heating the acetone solution to room temperature and removing the dioxygen under a vacuum (Figure S15). The transformation is nearly quantitative in both directions (>95% according to EPR data, Table S4). However, this equilibrium is strongly influenced by the solvent donor capacity. Thus, the behavior in the noncoordinating solvent 1,2-difluorobenzene is very similar to that just described for acetone with **4** formed at a low temperature and **2** recovered at room temperature (Figure S16). In contrast, coordination of the dioxygen molecule to the palladium center is promoted by a more coordinating solvent such as acetonitrile (Scheme 2b). In this solvent, the EPR spectrum of **2** under dioxygen shows only signals corresponding to the superoxide complex at both low and room temperatures (Figure S17a,b). Furthermore, complex **2** is not recovered even after removing the O_2_ atmosphere with several vacuum/argon cycles (Figure S17c).

These observations are related to the specific coordination requirements of palladium(I) and palladium(II) to form stable complexes. The relatively strong coordination of acetonitrile stabilizes the palladium(II) superoxido complex as a tetracoordinate adduct, **4**·NCMe. The weaker coordination of acetone to palladium(II) makes it less suitable for preventing reduction to palladium(I). Finally, the formation of the superoxido complex **4** in the noncoordinating solvent 1,2-difluorobenzene indicates that the three-coordinate structure shown in Scheme 2 is stable, at least at low temperatures.^11,17,31−33^

In conclusion, we successfully synthesized and thoroughly characterized the first air-stable palladium(I) mononuclear complex with an N-heterocyclic carbene ligand. Our findings confirm the metalloradical nature of the complex with the unpaired electron mainly residing in the palladium center. Moreover, we observed the reversible binding of dioxygen, leading to the formation of the three-coordinate superoxido palladium(II) complex [Pd(IPr)_2_(η^1^-OO^•^)][PF_6_] (**4**) at low temperatures. This reversibility is facilitated in weakly coordinating solvents by the low stability of the three-coordinate environment for Pd^II^. The availability of a vacant coordination position in **4** presents a compelling opportunity to regulate the reactivity of the superoxide group in oxidative transformations and specifically in hydrogen atom abstraction from C–H bonds in organic compounds.

## Safety Statement

No uncommon hazards are noted. See the SI for more details.

## Supporting Information Available

General experimental procedures; synthesis, cyclic voltammograms, and NMR, EPR, ESI-MS spectra for complexes **1** and **2**; experimental details, and NMR and EPR spectra for the formation of the superoxido complex **4**; procedure for the assignment of ^1^H NMR resonances; crystallographic data; Mulliken atomic spin densities for **2** and **4**, and other computational details (PDF)
