# Supplementary material for: Reversible Single-Electron-Transfer to Oxygen in a Stable N-Heterocyclic Carbene Palladium(I) Metalloradical
Source: Inorg Chem. 2023 Nov 22;62(49):19838–42. doi: 10.1021/acs.inorgchem.3c02878 (PMC10716904; doi:10.1021/acs.inorgchem.3c02878)
Supplement: Supplementary file 1 — ic3c02878_si_001.pdf [file ic3c02878_si_001.pdf]

Supporting Information

Reversible Single-Electron-Transfer to Oxygen in a Stable N-Heterocyclic Carbene Palladium(I) Metalloradical

Georgiana Maties, Pilar Gómez-Sal, Cristina G. Yebra*, Román Andrés*, and Ernesto de Jesús*

Departamento de Química Orgánica y Química Inorgánica, Instituto de Investigación Química "Andrés M. del Rio", Universidad de Alcalá, Campus Universitario, E28805 Alcalá de Henares, Madrid, Spain.

cgarcia.yebra@uah.es, roman.andres@uah.es, ernesto.dejesus@uah.es

Table of Contents

1. General Experimental Procedures **S3**

1.1. Synthetic Techniques S3

1.2. Instrumental Techniques S3

1.3. Safety Statment S4

2. Bis[1,3-bis(2,6-diisopropylphenyl)imidazol-2-ylidene]palladium(0), [Pd(IPr)$_2$] (1) **S5**

2.1. Synthesis S5

2.2. Characterization Data S5

2.3. ^{1}H and ^{13}C{^{1}H} NMR Spectra (Figures S1 and S2) S6

3. Bis-[1,3-bis(2,6-diisopropylphenyl)imidazol-2-ylidene]palladium(I) Hexafluoridophosphate, [Pd(IPr)$_2$][PF$_6$] (2) **S7**

3.1. Synthesis S7

3.2. Characterization Data S7

3.3. Mass Spectrum (Figure S3) S8

3.3. ^{1}H NMR Data and Spectra (Tables S1-S2 and Figures S4-S8) S8

3.4. EPR Data and Spectra (Table S3 and Figures S9-S10) S12

3.5. Stability of Complex **2** in Solution (Figure S11) S14

4. Reaction of Complex 2 with Dioxygen: Formation of the Superoxido Complex 4 **S16**

4.1. Evaluation of the Stability of Complex **2** in the Solid State in Air (Figure S12) S16

4.2. General Procedure for Reactions with Dioxygen in Solution S16

4.3. Reaction of **2** with Dioxygen in Acetone (Figures S13-S15 and Table S4) S16

4.4. Reaction of **2** with Dioxygen in 1,2-Difluorobenzene (Figure S16) S19

4.5. Reaction of **2** with Dioxygen in Acetonitrile (Figure S17) S20

4.6. EPR Data and Spectra of **4** in Frozen Solvents (Table S5 and Figure S18) S21

5. Cyclic Voltammetry Studies (Figures S19-S21) **S23**

6. Procedure for the Assignment of ^{1}H NMR Resonances **S25**

6.1. Complex **2** (Table S6 and Figure S22) S25

6.2. Complex **4** (Figure S23) S26

7. Procedure for the Magnetic Susceptibility Measurements (Figure S24) **S28**

8. Crystallographic Data **S29**

8.1. Selected Crystallographic Data of Complex **2** Compared to **1** (Table S7) S29

8.2. Crystal Structure Determination Details (Table S8 and Figure S25-S26) S30

9. Computational Details **S32**

9.1. Mulliken Atomic Spin Densities for Complexes **2** and **4** (Tables S9 and S10) S32

9.2. Computational Details (Table S11) S32

10. References **S36**

1. General Experimental Procedures

1.1. Synthetic Techniques

All operations were performed under an argon atmosphere using Schlenk or dry-box techniques. Tris(dibenzylideneacetone)dipalladium ($Pd_2(dba)_3$),[1,2] 1,3-bis(2,6-diisopropylphenyl)imidazolium chloride (IPr·HCl),[3,4] and ferrocenium hexafluoridophosphate ($[FeCp_2][PF_6]$)[5] were prepared according to literature procedures. Unless otherwise stated, all other reagents were obtained from commercial sources and used as received. 1,2-Difluorobenzene (Fluorochem, 98%) was distillated under argon and deoxygenated prior to use. Dimethyl sulfoxide (dmso) was dried by passing the solvent through a column of molecular sieve beads (4 Å, 4-8 mesh) under argon. Acetone and acetonitrile were purchased dry, deoxygenated, and stored over molecular sieves. Other solvents (HPLC grade) were purified by flash column chromatography and collected under argon, using a MBraun MB SPS solvent purification device.

1.2. Instrumental Techniques

NMR spectroscopy. NMR spectra were recorded using Varian Mercury 300, Unity 300, or Unity 500 Plus spectrometers. Chemical shifts (δ, parts per million) are quoted relative to $SiMe_4$ and were measured by internal referencing to the ^{13}C and residual ^{1}H resonances of the deuterated solvents. Coupling constants (J) are given in Hz.

EPR spectroscopy. CW-EPR spectra were performed in a Bruker EMX spectrometer at the C. A. I. de Resonancia Magnética Nuclear y de Espín Electrónico of the Universidad Complutense de Madrid. EPR data were obtained using a 20 mW microwave power in a HSW-10432 cavity at 9.46 GHz, with 4 G field modulation at 100 kHz. For measurements in frozen solutions, the complex was dissolved in 2 mL of the appropriate solvent (molar concentration of 10^{-3} M) and the solution was quickly frozen with liquid nitrogen. EPR spectra were simulated using the software EasySpin[6,7] in combination with the SimLabel graphical user interface.[8]

Cyclic Voltammetry. Cyclic voltammograms (CV) were recorded with a CH Instrument model 620E series potentiostat using a homemade cell fitted with a Pt working electrode (CH Instruments CHI102, diameter = 2 mm), a Pt wire counter electrode (CHI115, diameter = 0.5 mm, length = 32 mm), and an Ag wire pseudoreference electrode (CHI112). Measurements were performed using solutions of the complex (2 mM) and electrolyte ($[N^nBu_4][PF_6]$, 0.2 M) in 1,2-difluorobenzene. All potentials are given relative to the ferrocenium/ferrocene couple ($[Fc]^+/Fc$) that was used as an internal standard. After recording the cyclic voltammogram, the internal standard (ferrocene, 4 mM) was added to the solution and the CV recorded again.

Mass spectrometry. Mass spectra were recorded at the Analytical Services of the Universidad de Alcalá using an Agilent 6210-TOF LC/MS mass spectrometer with an ESI ion source. After

dissolution in the appropriate solvent, the samples were directly pumped into the mass spectrometer and analyzed without additives.

Elemental analyses. The Analytical Services of the Universidad de Alcalá performed the C, H, and N analyses using a LECO CHNS-932 microanalyzer.

1.3 Safety Statement

The procedures outlined in this report have been described so that they can be safely reproduced by personnel trained in the common hazards associated with chemical synthesis (use of flammable solvents, handling of cryogenic liquids, use of vacuum lines, etc.). All volatile substances should be handled in a fume hood. Glass containers with pressurized gases must be protected with a protective screen. The glass material used in this work may not be safe at pressures higher than those reported here. No uncommon hazards are noted following the procedures described in this work, provided they are performed by trained personnel on the scale reported herein.

2. Bis[1,3-bis(2,6-diisopropylphenyl)imidazol-2-ylidene]palladium(0), [Pd(IPr)$_2$] (1)

2.1. Synthesis

The preparation of this complex has been previously reported by reduction of [Pd(η^3-C_3H_5)(Cl)(IPr)] with KO*t*Bu in the presence of an equivalent of IPr ligand.[9] The complete preparative process starting from the accessible Pd salt Na_2PdCl_4 has an overall 74% yield on palladium and takes around 6 working days.[9-11] The direct displacement of dba by NHC from the accessible and relatively air-stable complex $Pd_2(dba)_3$ (Scheme S1) has been rarely used for the synthesis of [Pd(NHC)$_2$] complexes because the observation of side reactions between the displaced dba and the free carbene.[12] Nevertheless, complex **1** can actually be obtained by this method in an analytically and spectroscopically pure form following the procedure reported bellow. The whole preparative process from $PdCl_2$ has an overall 75% yield and takes around 2 working days.

$Pd_2(dba)_3$ (0.500 g, 0.546 mmol) and 1,3-bis(2,6-diisopropylphenyl)imidazolium chloride (0.928 g, 2.18 mmol) were stirred for 4 h under vacuum in dry dmso (5 mL) in presence of NaO*t*Bu (0.210 g, 2.18 mmol). Complex **1** was isolated as an orange-yellow solid by filtration, washed with acetone (10 mL) and dried under vacuum (0.717 g, 75 %).

Scheme S1. Synthesis of Complex [Pd^0(IPr)$_2$] (1)

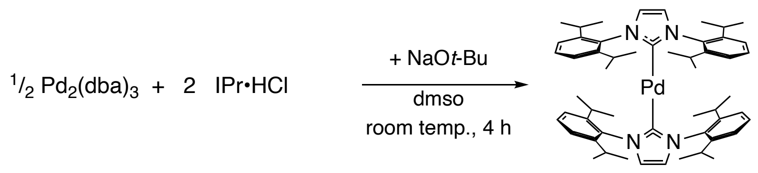

1

2.2. Characterization Data

Anal. Calcd for $C_{54}H_{72}N_4Pd$: C, 73.40; H, 8.21; N, 6.34. Found: C, 72.93; H, 7.86; N, 6.80. ^{1}H NMR (300 MHz, C_6D_6, 7.16 ppm): δ 7.30 (t, J = 7.7, 4H, Ar-*p*-C*H*), 7.09 (d, J = 7.7, 8H, Ar-*m*-C*H*), 6.28 (s, 4H, Imz-C*H*), 2.90 (sept, J = 6.9, 8H, C*H*Me$_2$), 1.23 (d, J = 6.9, 24H, CH*Me*$_2$), 1.13 (d, J = 6.9, 24H, CH*Me*$_2$). ^{13}C{^{1}H} NMR (75 MHz, C_6D_6, 128.06 ppm): δ = 199.4 (Imz-C^2), 146.3 (Ar-C^2), 139.4 (Ar-C^1), 128.9 (Ar-C^4), 123.7 (Ar-C^3), 121.6 (Imz-C^{4,5}), 29.2 (*C*HMe$_2$), 25.7 (*C*H$_3$), 24.6 (*C*H$_3$).

2.3. ^{1}H and $^{13}C\{^{1}H\}$ NMR Spectra (Figures S1 and S2)

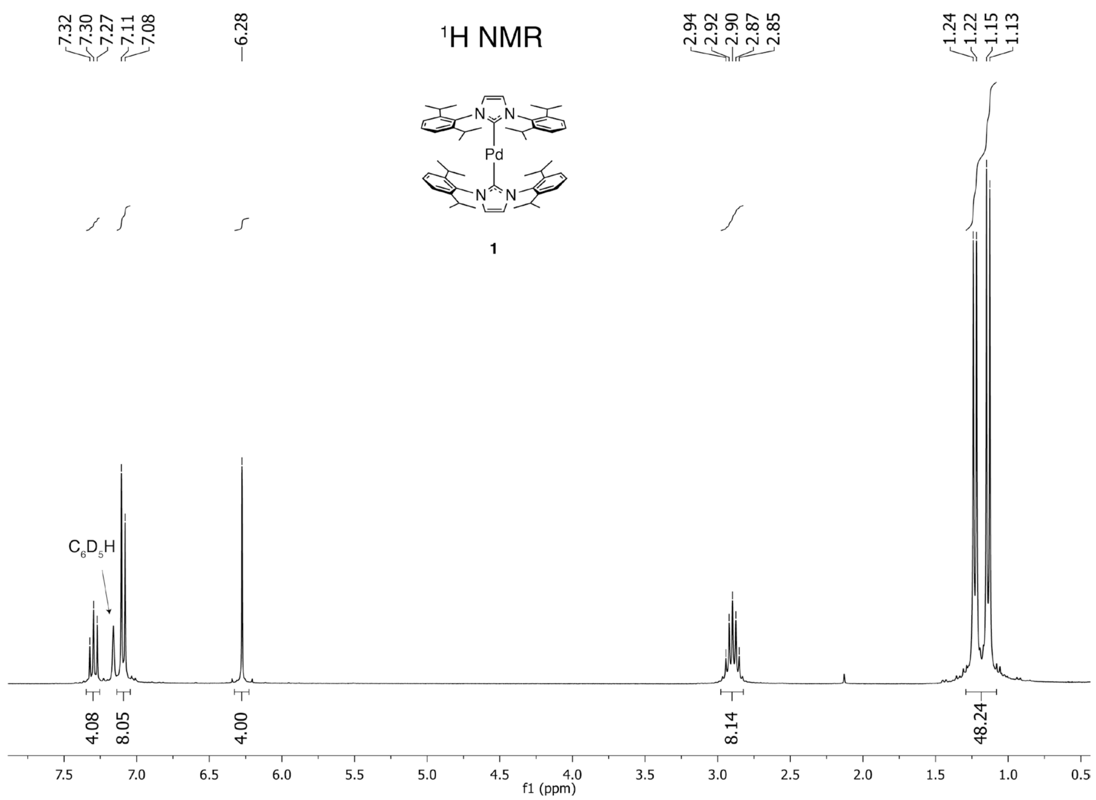

Figure S1. ^{1}H NMR spectrum (300 MHz) for **1** in benzene-d_6.

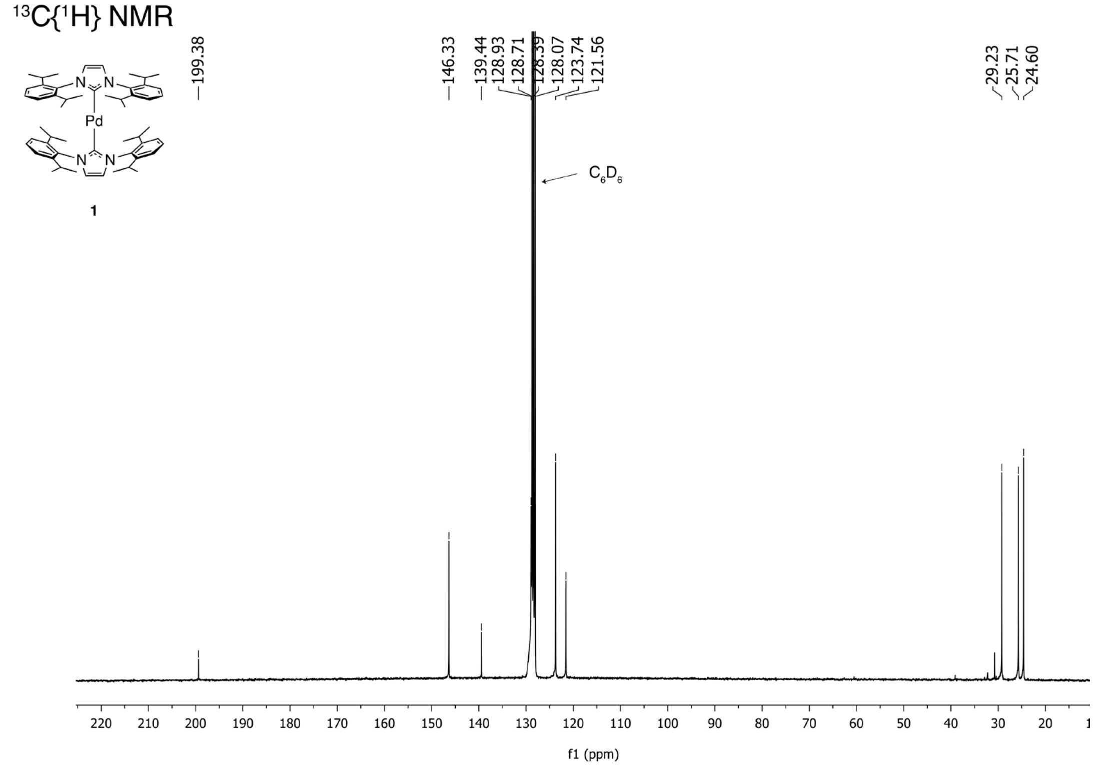

Figure S2. $^{13}C\{^{1}H\}$ NMR spectrum (75 MHz) for **1** in benzene-d_6.

3. Bis-[1,3-bis(2,6-diisopropylphenyl)imidazol-2-ylidene]palladium(I) Hexafluoridophosphate, [Pd(IPr)$_2$][PF$_6$] (2)

3.1. Synthesis

A mixture of complex **1** (0.640 g, 0.724 mmol) and ferrocenium hexafluoridophosphate (0.240 g, 0.724 mmol) in 1,2-difluorobenzene (6 mL) was stirred for 2 h at room temperature (Scheme S2). After this period, the solution was filtered through a celite pad under inert atmosphere and hexane (10 ml) was then added to the filtrate. The precipitate thus obtained was filtered, washed with hexane (5 mL) to remove ferrocene, and dried in vacuo to give complex **2** as a pale yellow solid (0.520 g, 70%).

Scheme S2. Synthesis of Complex [Pd(IPr)$_2$][PF$_6$] (2)

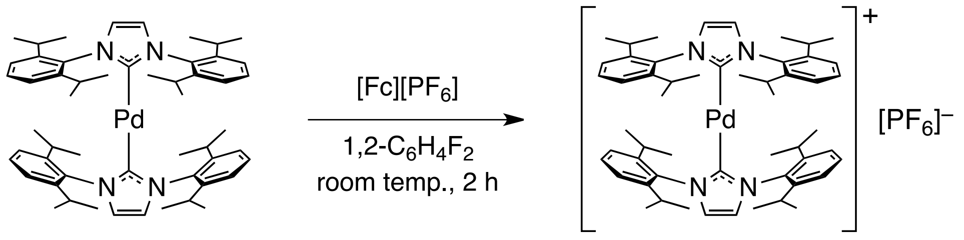

1 **2**

Complex **2** is stable for an indefinite period in air in the solid state and can be handled without special precautions. The complex is stable for at least several days in solution at room temperature under inert atmosphere but decomposes more rapidly under dioxygen.

3.2. Characterization Data

Anal. Calcd for $C_{54}H_{72}F_6N_4PPd$: C, 63.06; H, 7.06; N, 5.45. Found: C, 63.41; H, 7.10; N, 5.47. ^{1}H NMR (300 MHz, CD_2Cl_2, 5.32 ppm, 25 °C): δ 18.46 ($\Delta\nu_{1/2}$ = 320 Hz, 4H, Imz-C*H*), 8.62 ($\Delta\nu_{1/2}$ = 64 Hz, 8H,Ar-*m*-C*H*), 6.99 ($\Delta\nu_{1/2}$ = 723 Hz, 24H, CH*Me*$_2$), 6.90 ($\Delta\nu_{1/2}$ = 41 Hz, 4H, Ar-*p*-C*H*), 4.12 ($\Delta\nu_{1/2}$ = 96 Hz, 24H, CH*Me*$_2$), 0.98 ($\Delta\nu_{1/2}$ = 485 Hz, 8H, C*H*Me$_2$). HR ESI-MS (positive ion, 1,2-difluorobenzene): *m*/*z* 882.4781 (calcd for [**2** − PF$_6$]$^+$, $C_{54}H_{72}N_4Pd^+$ 882.4810). Effective magnetic moment (Evans method, 90:10 v/v CD_2Cl_2/cyclohexane solution, 298 K): μ_{eff} 1,68 μ_B.

3.3. Mass Spectrum (Figure S3)

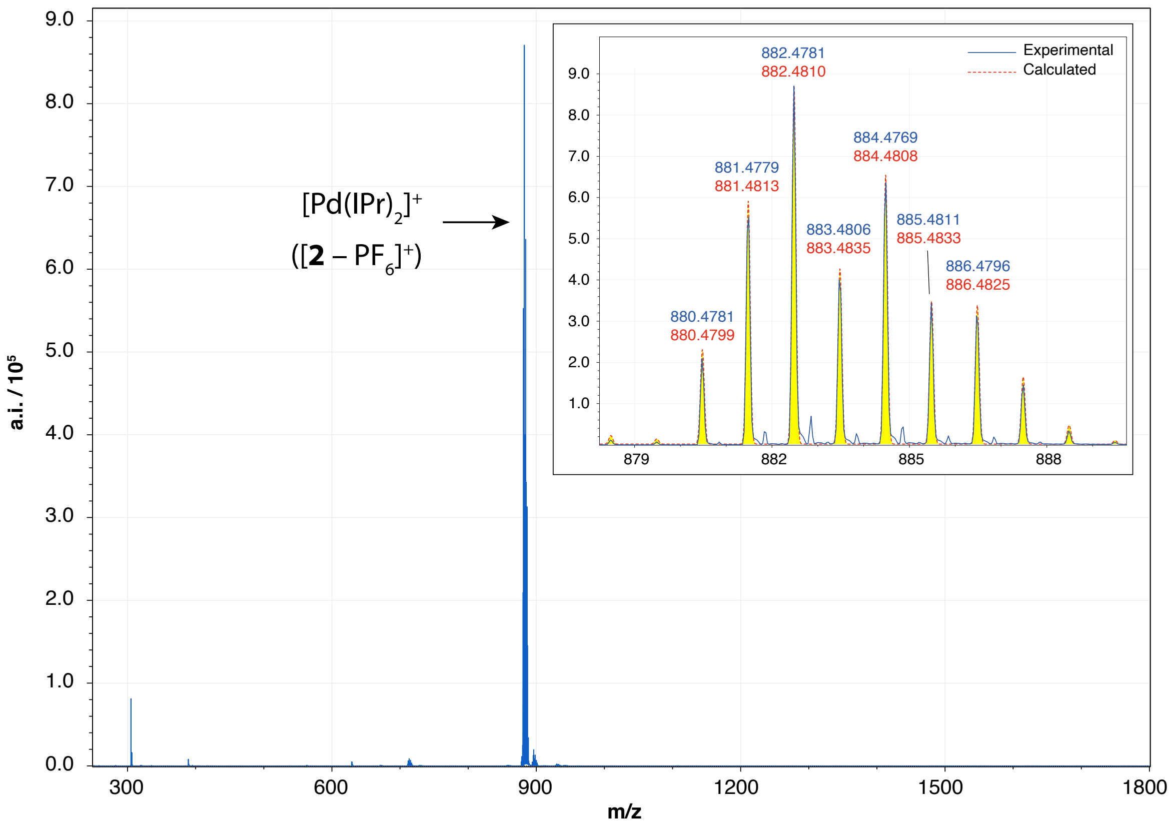

Figure S3. ESI-TOF(+) mass spectrum of **2** in 1,2-difluorobenzene. The inset shows an enlarged view of the experimental (blue lines and digits) and calculated peaks (red lines and digits) corresponding to the $[Pd(IPr)_2]^+$ molecular ion.

3.3. ¹H NMR Data and Spectra (Tables S1-S2 and Figures S4-S8)

Table S1. ¹H NMR Chemical Shifts (δ, Ppm) for Complex 2 in Different Solvents (298 K)

Assignment	CD_2Cl_2	Acetone-*d*6	CD_3CN	THF-*d*8
Imz-C*H*	18.46	18.97	18.90	18.49
Ar-*m*-C*H*	8.62	8.76	8.68	8.67
Me(1)	6.99	6.90	6.87	7.15
Ar-*p*-C*H*	6.90	6.75	6.74	6.85
Me(2)	4.12	4.03	3.91	4.18
C*H*Me2	0.98	1.08	0.97	--[a]

[a] This resonance could not be located.

Table S2. Variable-Temperature ^{1}H NMR Data for Complex 2 in CD_2Cl_2

Chemical shifts (δ, ppm)										
T (K)	308.15	298.15	283.15	273.15	263.15	253.15	243.15	233.15	223.15	213.15
Imz-C*H*	18.17	18.46	19.10	19.57	20.08	20.62	21.25	21.88	22.60	23.45
Ar-*m*-C*H*	8.60	8.62	8.67	8.72	8.76	8.82	8.87	8.93	9.00	9.09
Me(1)	6.90	6.99	7.18	7.36	7.56	7.84	8.16	8.41	8.69	9.06
Ar-*p*-C*H*	6.91	6.90	6.87	6.85	6.83	6.81	6.79	6.77	6.75	6.74
Me(2)	4.07	4.12	4.24	4.33	4.43	4.55	4.67	4.80	4.96	5.15
C*H*Me$_2$	1.00	0.98	0.80	0.70	0.59	0.47	0.40	0.25	0.25	0.10
Linewidths ($\Delta\nu_{1/2}$, Hz)										
T (K)	308.15	298.15	283.15	273.15	263.15	253.15	243.15	233.15	223.15	213.15
Imz-C*H*	331	320	415	410	493	540	610	693	819	929
Ar-*m*-C*H*	60	64	78	89	103	118	134	156	182	212
Me(1)	741	723	1022	1159	1339	1800	2125	2186	2882	3556
Ar-*p*-C*H*	37	41	48	53	61	71	80	96	110	133
Me(2)	93	96	114	124	137	151	171	196	218	253
C*H*Me$_2$	479	485	668	765	835	861	1071	1157	1437	1570

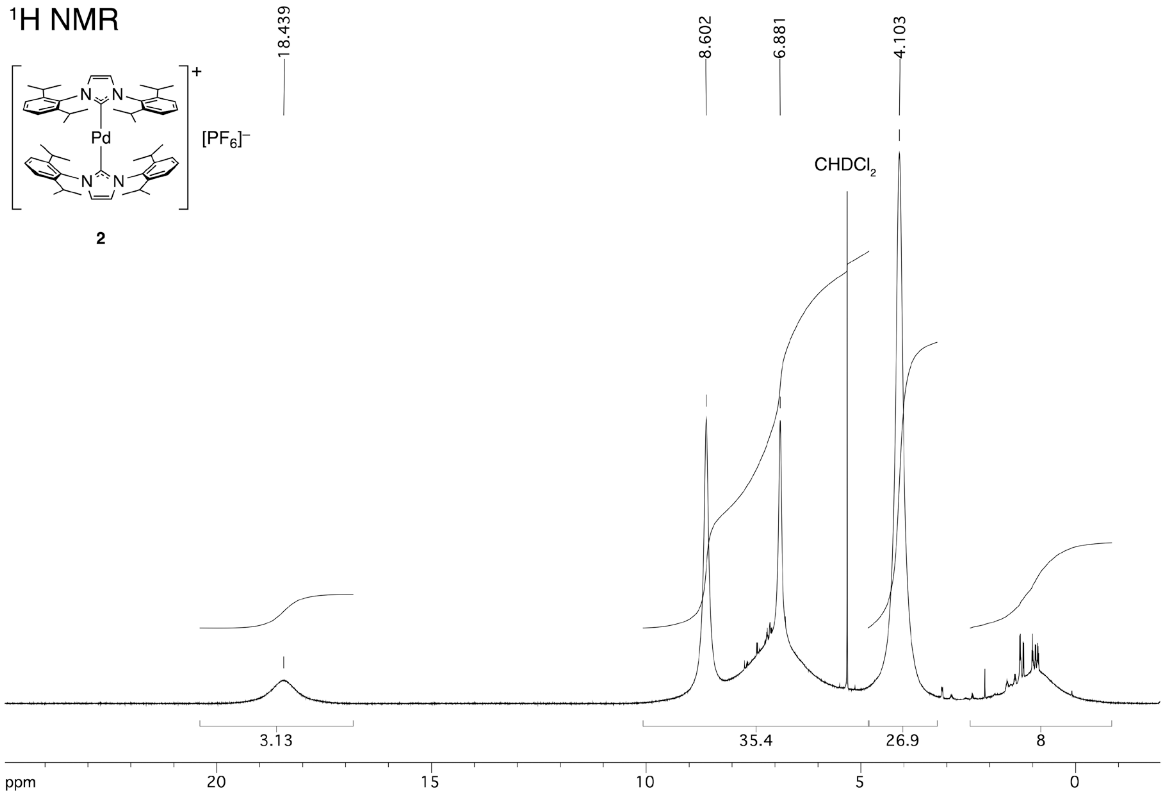

Figure S4. ^{1}H NMR spectrum (300 MHz) for **2** in CD_2Cl_2 at 298.3 K.

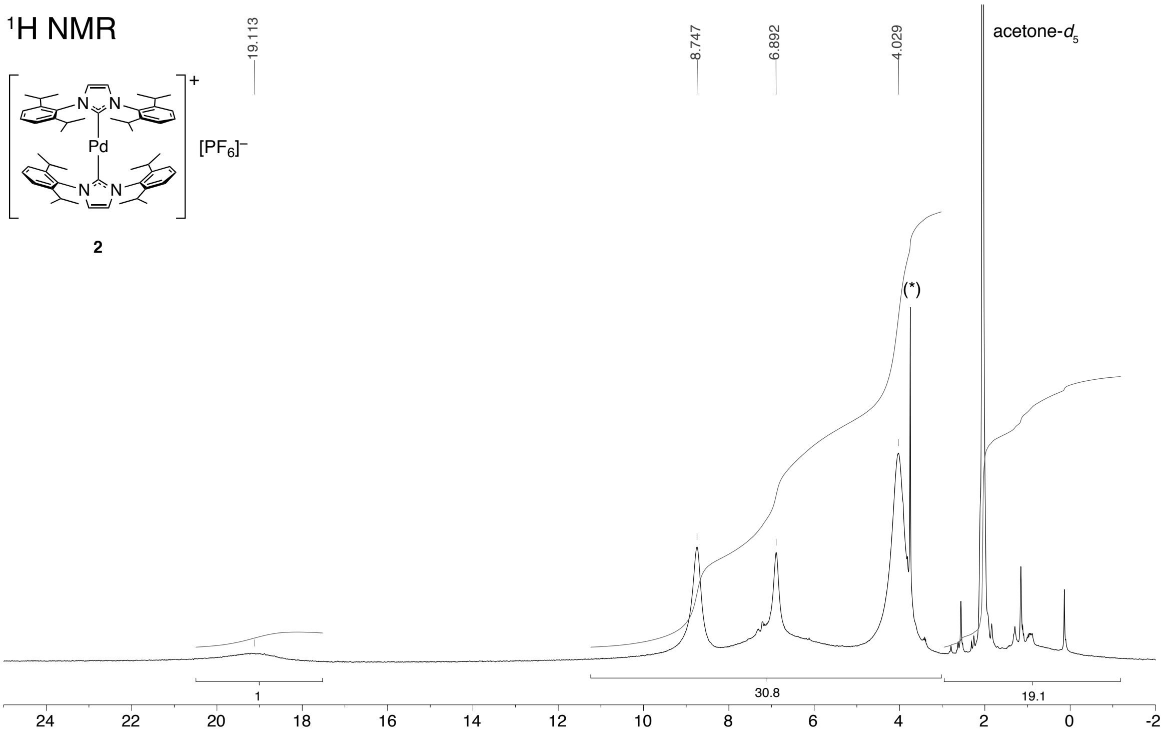

Figure S5. ^{1}H NMR spectrum (500 MHz) for **2** in acetone-d_6 at 298.3 K. (*): impurity proceeding from the deuterated solvent.

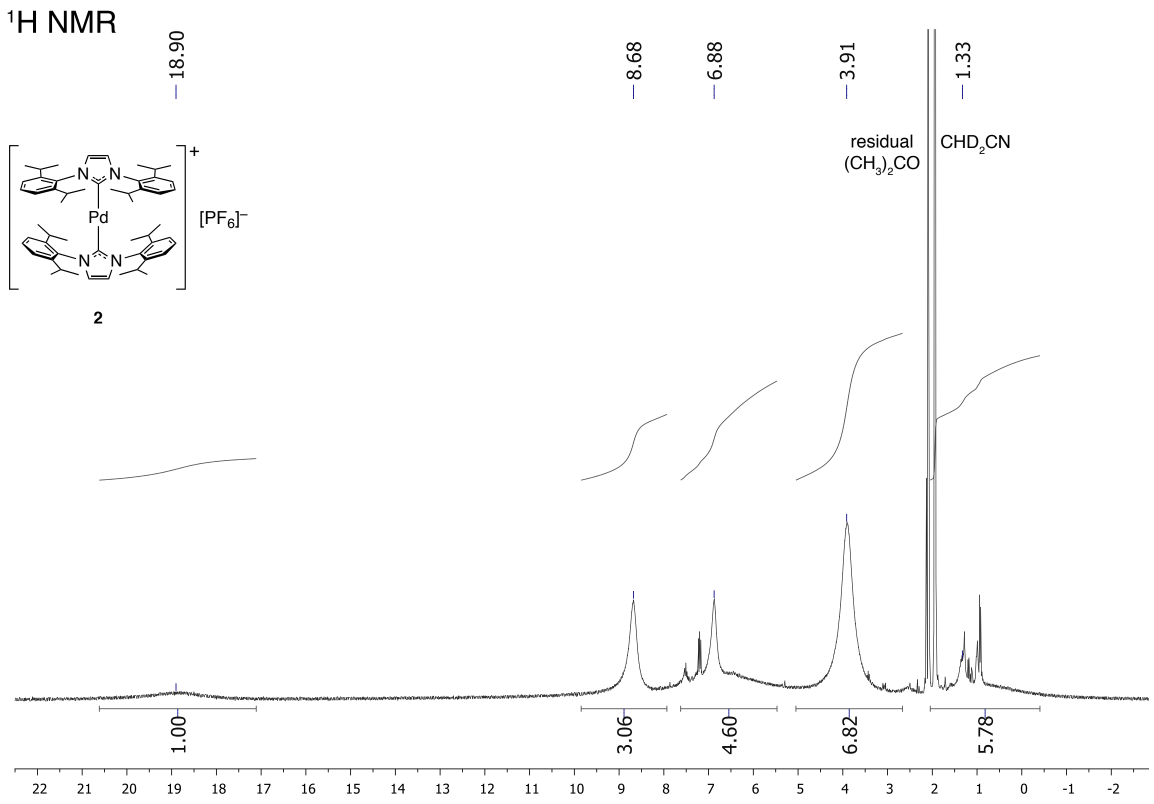

Figure S6. ^{1}H NMR spectrum (500 MHz) for **2** in CD_3CN at 298.3 K.

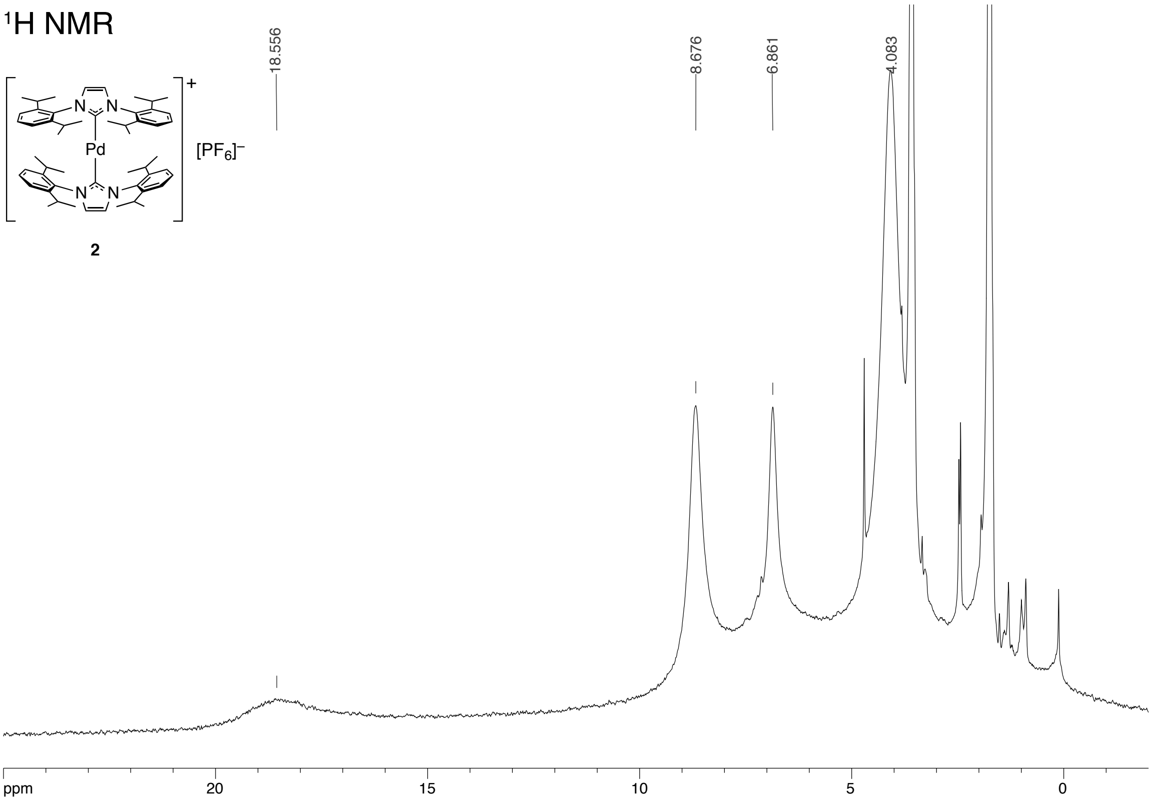

Figure S7. ^{1}H NMR spectrum (500 MHz) for **2** in THF-d_8 at 298.3 K.

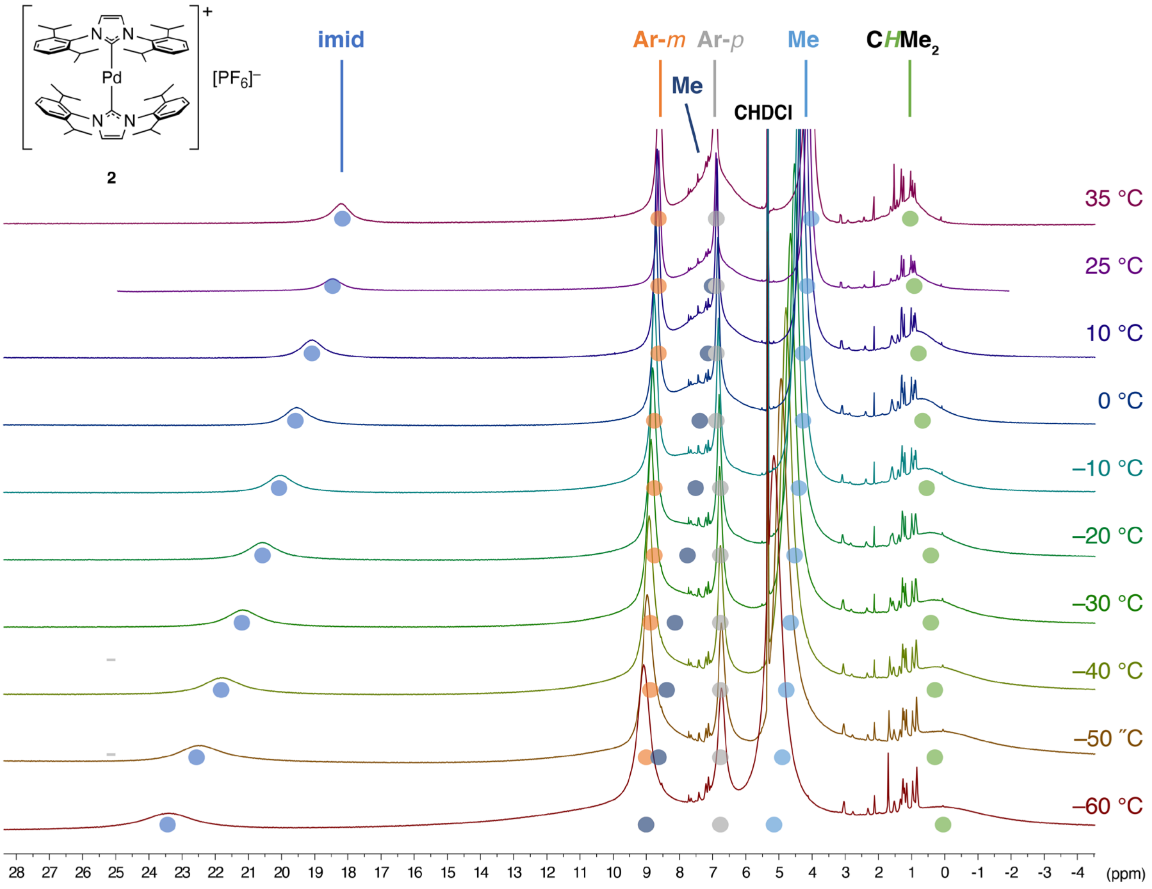

Figure S8. Variable-temperature ^{1}H NMR spectra (500 MHz) for **2** in CD_2Cl_2.

3.4. EPR Data and Spectra (Table S3 and Figures S9-S10)

Table S3. EPR Data for Complex 2 in Solid State and Frozen Solvents

Media	Temperature (K)	$g_{\parallel}$	$g_{\perp}$	$A_{\parallel}$ (mT)	$A_{\perp}$ (mT)
1,2-Difluorobenzene	200 K	1.973	2.385	24.9	25.2
Acetonitrile	200 K	1.983	2.381	25.3	25.3
Acetone	170 K	1.973	2.384	27.4	25.5
Solid	200 K	1.969	2.401	-	-

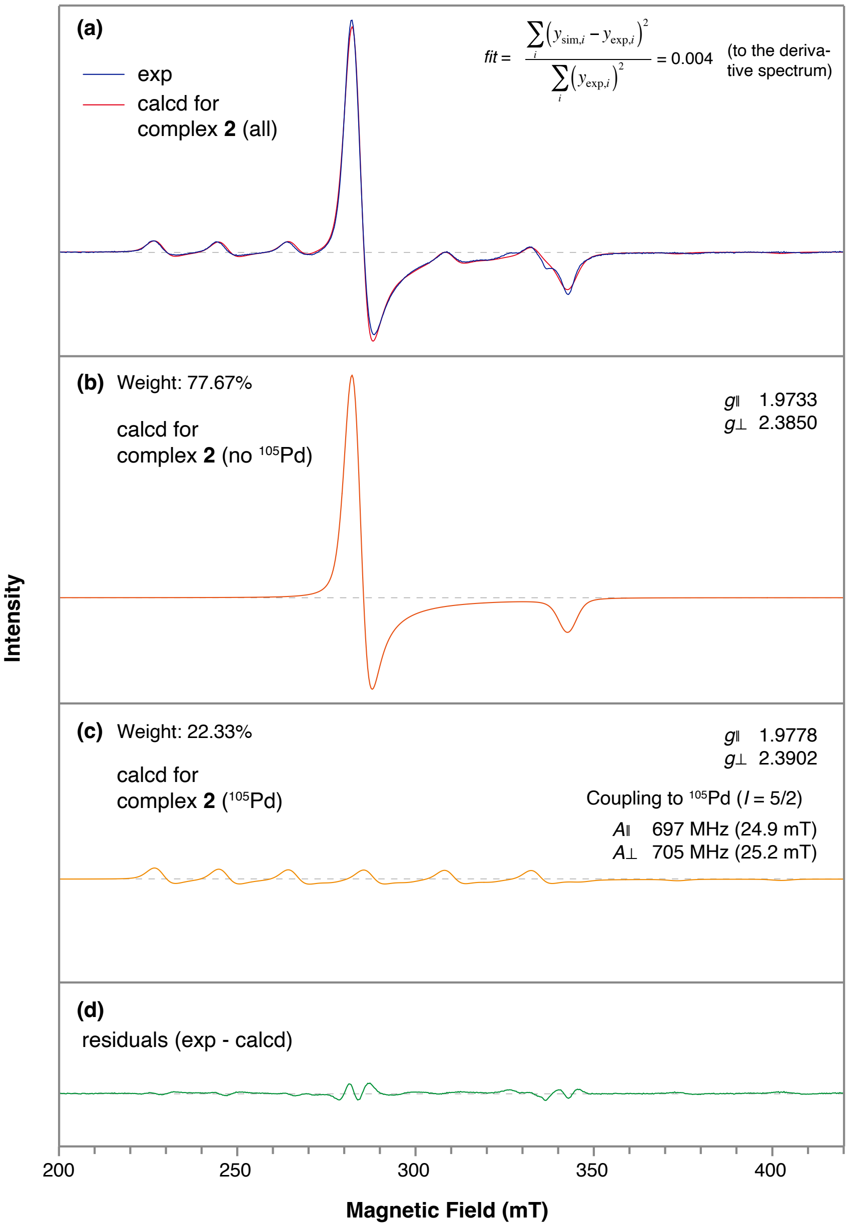

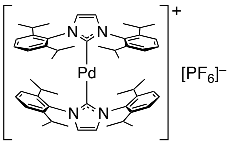

2

Figure S9. (a) Experimental (blue line) and calculated (red line) EPR spectra of a frozen solution of complex **2** in 1,2-difluorobenzene at 200 K. (b) Calculated spectrum and simulation parameters for the non ^{105}Pd isotopologues. (c) Calculated spectrum and simulation parameters for the ^{105}Pd isotopologue. (d) Residuals (experimental-calculated spectra).

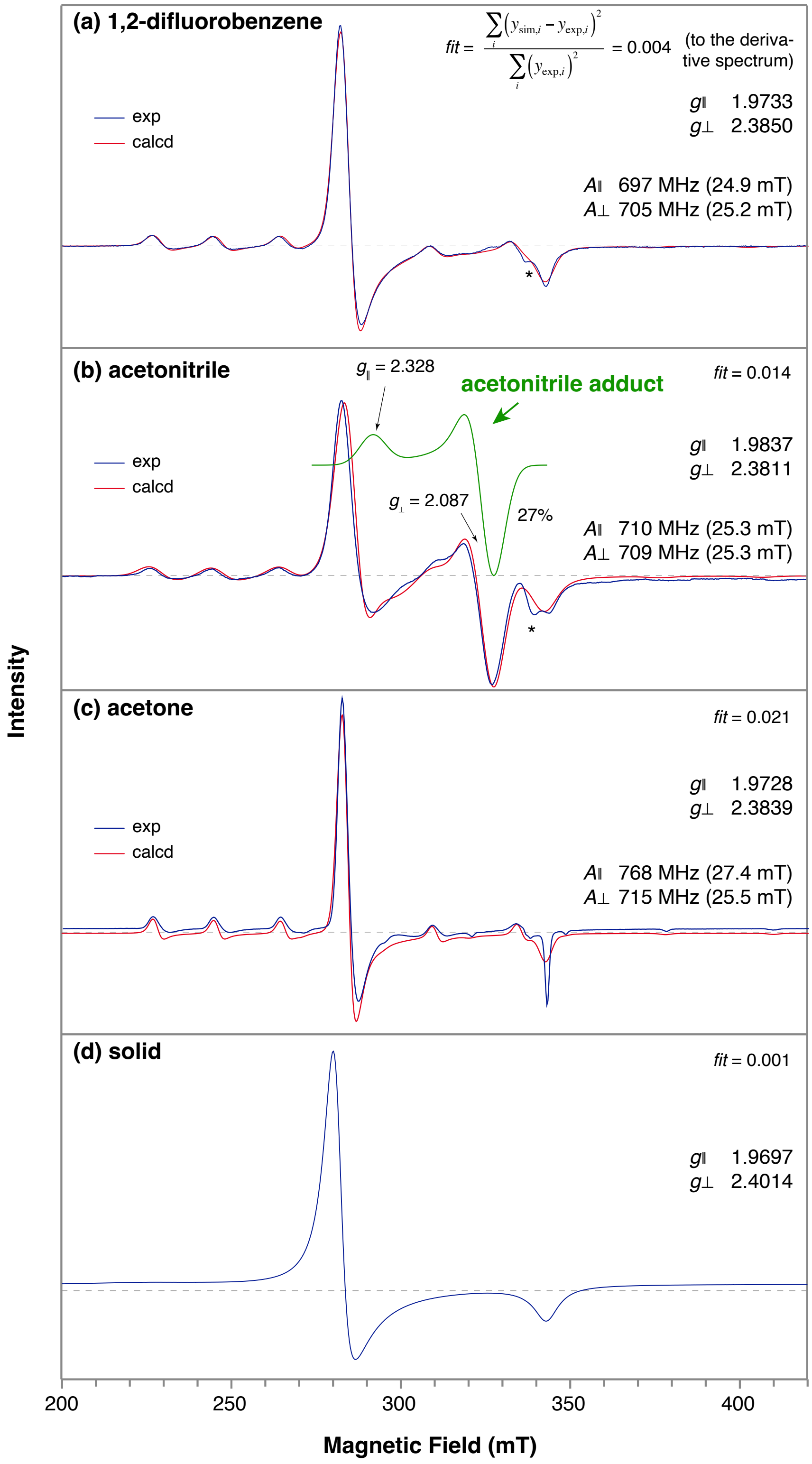

$$fit = \frac{\sum_i \left(y_{\mathrm{sim},i} - y_{\mathrm{exp},i}\right)^2}{\sum_i \left(y_{\mathrm{exp},i}\right)^2}$$

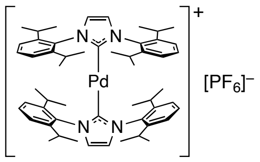

2

Figure S10. Experimental and fitted EPR spectra of complex **2** in (a) 1,2-$C_6H_4F_2$ at 200 K, (b) acetonitrile at 200 K, and (c) acetone at 170 K. (d) EPR spectrum of complex **2** in solid state at 200 K. In the case of the spectrum in acetonitrile (b), an acceptable correspondence between the experimental and calculated spectra was only obtained when a 27% of contribution of a second component was considered in the simulation. This component, the contribution of which is represented in the drawing by a **green line**, is attributed to the partial formation of the acetonitrile adduct $[Pd(IPr)_2(NCCH_3)][PF_6]$ (**3**). The asterisk-labeled shoulder indicates the production of a minor quantity of the superoxido complex **4**.

Atomic Spin Density from Hyperfine Couplings. We have deduced the atomic spin density on palladium from the ^{105}Pd hyperfine constant values determined by fitting the frozen solution EPR spectra of complex **2** show in Figure S10.[13,14] For this purpose, we have chosen the spectrum obtained in acetone (Figure S10c), since it shows more defined signals and allows a more accurate estimation of the hyperfine couplings with palladium-105. We have used the atomic parameters compiled by Koh and Miller to obtain the spin densities from the isotropic and anisotropic contributions to the hyperfine coupling constant.[14]

The hyperfine coupling values determined for complex **2** by fitting the spectrum obtained in frozen acetone are $A_{\parallel} = 768$ MHz and $A_{\perp} = 715$ MHz. The isotropic (A_{iso}) and anisotropic (T) terms are then

$$A_{iso} = {}^{1}/_{3} \times (768 + 2 \times 715) = 733 \text{ MHz}$$
$$T = {}^{1}/_{3} \times (768 - 715) = 18 \text{ MHz}$$

The isotropic hyperfine coupling for an electron occupying a 5*s* $^{105}Pd^{+}$ orbital can be calculated from the appropriate atomic parameters in ref [14] as:

$$A_1 = ({}^{8\pi}/_{3}) \times 24.50 \times 8 = 1642 \text{ MHz}$$

The value of 8 for the $|\psi(0)|^2$ function of a 5*s* orbital in a Pd^{+} ion is a rough estimate from values for neighboring atoms. Therefore, the estimate should be taken with caution.

The anisotropic hyperfine couplings for an electron occupying a 4*d* $^{105}Pd^{+}$orbital can be calculated from the appropriate atomic parameters in ref [14] as:

$$A_{2,zz} = ({}^{2}/_{21}) \times 24.50 \times 7.19 \times 6 = 101 \text{ MHz}$$

$$A_{2,xx} = A_{2,yy} = ({}^{2}/_{21}) \times 24.50 \times 7.19 \times 3 = 50 \text{ MHz}$$

The spin densities in the 5*s* and 4*d* Pd orbitals deduced from these hyperfine couplings are:

5*s*: (733 / 1642) *100 = **45%** (DFT: 27%)
4*d*: (18 / 50) *100 = **36%** (DFT: 60%)
overall on Pd: 45% + 35% = **81%** (DFT: 89%)

The spin density deduced from EPR hyperfine couplings agrees with the conclusion that the missing electron is essentially located at the palladium atom, although the contribution of the 5*s* and 4*d* orbitals to the SOMO differs notably from that calculated by DFT.

3.5. Stability of Complex 2 in Solution (Figure S11)

We have studied the stability of complex **2** in solutions of acetone-d_6, dichloromethane-d_2, chloroform-d_1, and acetonitrile-d_3 at room temperature by ^{1}H NMR spectroscopy. The ^{1}H NMR spectra show almost no changes after 72 h in dichloromethane, acetone, or acetonitrile. In contrast, a noticeable evolution is observed in chloroform. In this solvent, complex **2** evolves completely to a

mixture of diamagnetic compounds in about 11 days (Figure S11). This mixture is composed of the imidazolium cation $[IPr{\bullet}H]^+$, the dihalide $[Pd^{II}(IPr)_2Cl_2]$ and another unidentified IPr Pd^{II} complex. The reaction with the solvent is the most likely explanation for the formation of the chloride complex.

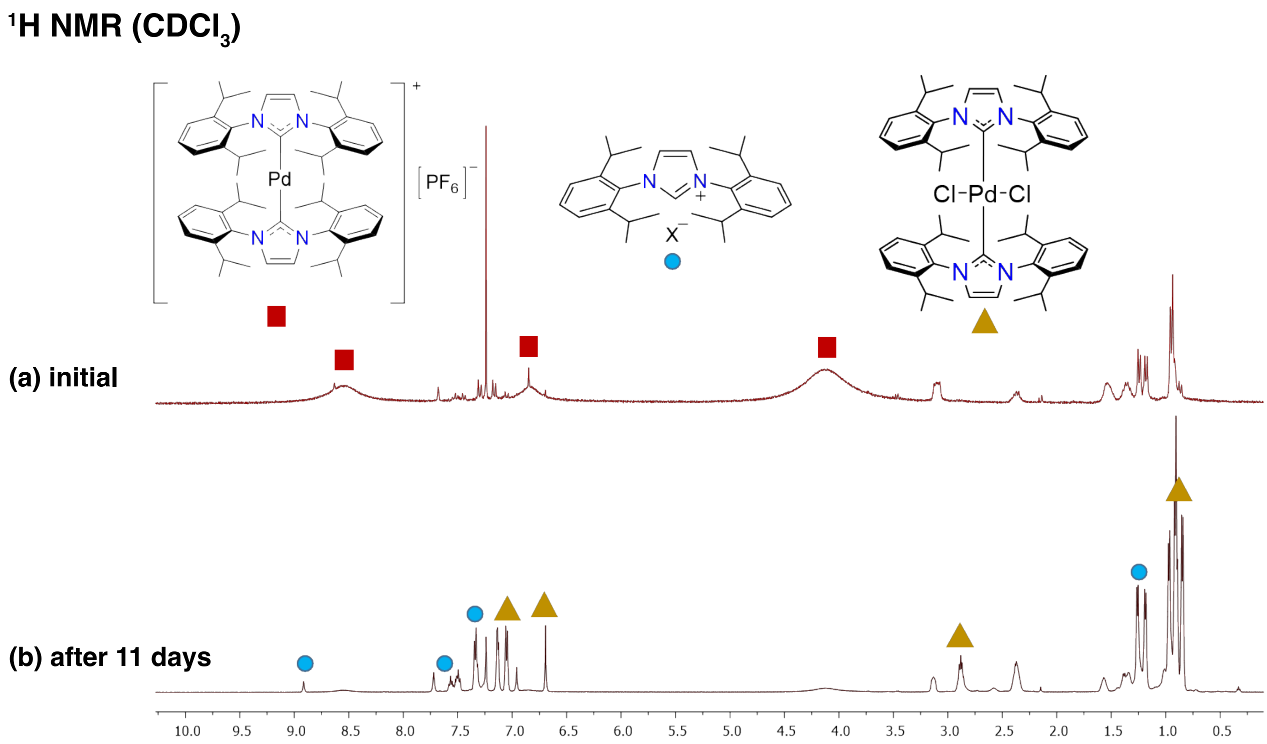

Figure S11. ^{1}H NMR spectra of complex **2** in $CDCl_3$ (a) immediately after dissolution and (b) after 11 days in solution at room temperature under argon.

4. Reaction of Complex 2 with Dioxygen: Formation of the Superoxido Complex 4

4.1. Evaluation of the Stability of Complex 2 in the Solid State in Air (Figure S12)

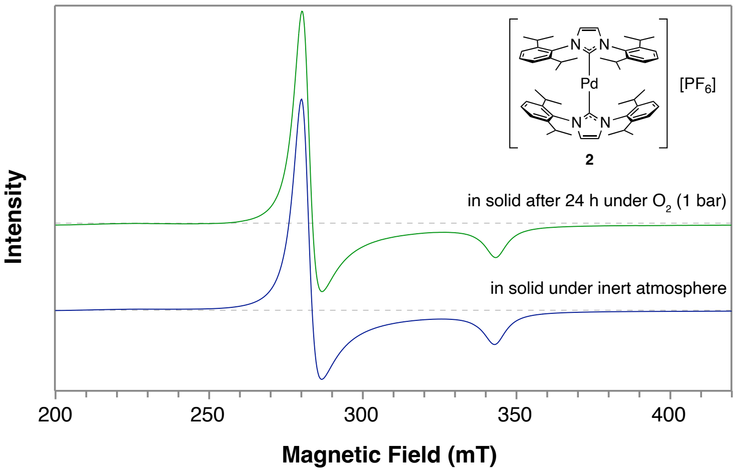

Figure S12. Comparison of EPR spectra of complex **2** before and after being subjected in solid to a dioxygen atmosphere (1 bar) for 24 hours. Both spectra were recorded at 200 K and are perfectly superimposable.

4.2. General Procedure for Reactions with Dioxygen in Solution

The experiments were performed either in valved NMR or EPR tubes. For ^{1}H NMR spectroscopy monitoring, complex **2** (9×10^{-6} mol) was dissolved under inert atmosphere in 0.6 mL of dry and deoxygenated deuterated solvent and the ^{1}H NMR spectrum was subsequently recorded. The valved tube was then pressurized with 1 bar of O_2 and the ^{1}H NMR spectrum recorded again at the desired temperature. To check the reversibility of the dioxygen addition, the solvent was removed under vacuum and the tube refilled with deuterated solvent before recording the ^{1}H NMR spectrum again.

For EPR spectroscopy monitoring, 2 mL of a $\approx 10^{-3}$ M solution of complex **2** were introduced in the valved tube which was subsequently pressurized with 1 bar of O_2. The solutions were cooled in the EPR cavity until freezing of the solvent. To check the reversibility of the dioxygen addition, the solvent was removed under vacuum at room temperature and the tube refilled with solvent before recording the EPR spectrum again.

4.3. Reaction of 2 with Dioxygen in Acetone (Figures S13-S15 and Table S4)

^{1}H NMR study. A solution of complex **2** in acetone-d_6 (*ca.* 1.5×10^{-2} M) was prepared in a valved NMR tube as described in Section 4.2. Variable-temperature ^{1}H NMR spectra of this solution were recorded every 5 °C between 25 °C and −80 °C (see Figure S13a for the spectrum at −10 °C). The valved tube was then pressurized with 1 bar O_2 at room temperature and ^{1}H NMR spectra were again recorded over the same temperature range (Figure S13b–d). Resonances for the new superoxido

complex **4** were clearly visible at −40 °C (Figure S13c). The spectra obtained at the same temperature in the decrease from 25 °C to −80 °C and in the ascent from −80 °C to 25 °C were almost identical, demonstrating that the conversion of **2** into **4** is reversible with temperature.

^{1}H NMR Data for 4 at 213 K . ^{1}H NMR (500 MHz, acetone-d_8, 2.05 ppm, 213 K): δ 7.57 ($\Delta\nu_{1/2}$ = 442 Hz, Int ≈ 14), 7.08 ($\Delta\nu_{1/2}$ = 362 Hz, Int ≈ 8), 2.26 ($\Delta\nu_{1/2}$ = 595 Hz, Int ≈ 14), 1.05 ($\Delta\nu_{1/2}$ = 174 Hz, Int ≈ 16), 0.83 ($\Delta\nu_{1/2}$ = 161 Hz, Int ≈ 20).

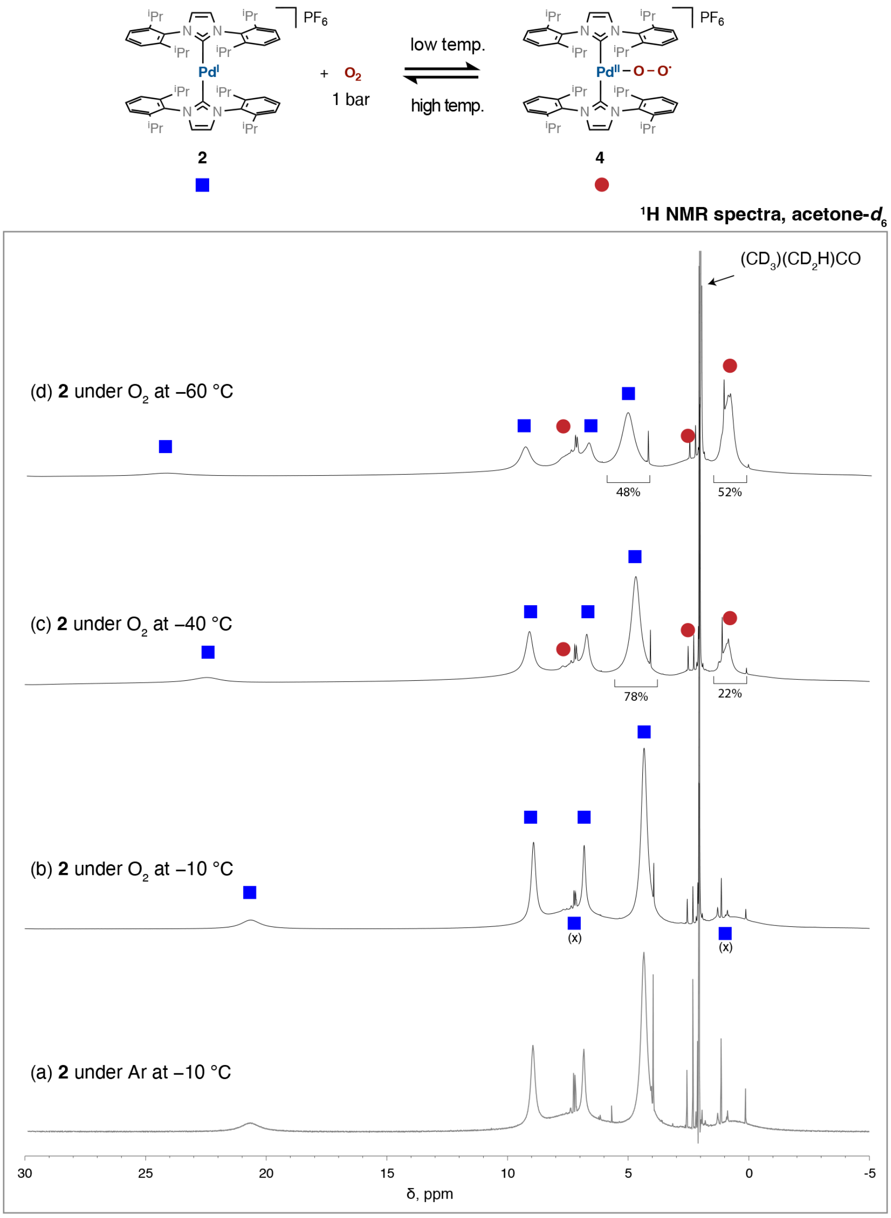

Figure S13. ^{1}H NMR spectra of the Pd(I) complex **2** at variable temperature in acetone-d_6 under an atmosphere of O_2 (1 bar). (a and b) Comparison of the spectra of complex **2** at −10 °C under argon and dioxygen, respectively. The resonances of **2** are identified with blue squares. No reaction with O_2 is observed at this temperature. (c) Three new resonances emerge at around 7.3, 2.5, and 0.9 ppm (red circles) at −40 °C under O_2. These resonances are assigned to the superoxido complex **4**. The major product at this temperature is complex **2** (the two resonances marked with a cross (×) in the spectrum b cannot clearly differentiated in spectra c and d). (d) The intensity of the resonances of the superoxide complex **4** increase notably at −60 °C.

EPR study. A valved EPR tube containing a solution of complex **2** in acetone was pressurized with 1 bar of O_2 as described in Section 4.2. The pressure was kept constant, at approximately 1 bar of total oxygen pressure, by connecting the tube valve to a balloon slightly inflated with oxygen. EPR spectra were then recorded every 10 °C between 25 °C and −85 °C. The signal of the superoxido complex **4** appeared clearly in the EPR spectra at 5 °C. The spectra obtained at the same temperature in the decrease from 25 °C to −80 °C and in the ascent from −80 °C to 25 °C were almost identical (see the spectra obtained at −15 °C in Figure S14). The signal of superoxido **4** became the only resonance observed between −55 °C and −65°C.

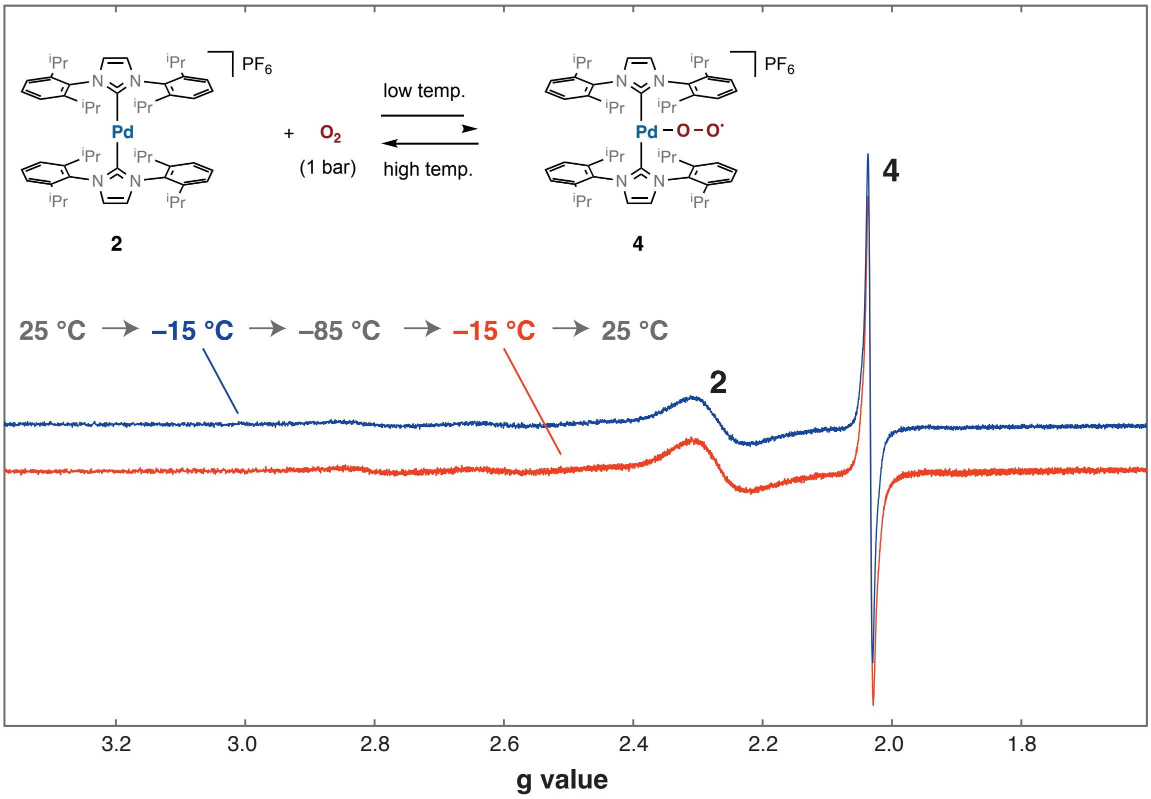

Figure S14. EPR spectra of the Pd(I) complex **2** in acetone under an atmosphere of O_2 (1 bar) at –15 °C. In blue, the spectrum obtained during the temperature drop from 25 to –85 °C. In red, the spectrum obtained during the subsequent temperature rise from –85 °C to 25 °C.

The concentrations of spin in the above EPR spectra were calculated using the quantification tool of Bruker's ESR Studio. The concentrations determined for several of the spectra are given in Table S4 in percent with respect to the spin concentration in the initial solution recorded at 25 °C. The spin concentration remains nearly constant during the gradual transformation of **2** into **4** that follows the temperature decrease, indicating a nearly quantitative transformation. In the reverse process, when the temperature is again increased, the spin concentration decreases slightly, likely due to the lower stability of the superoxide radical **4** compared to that of the metalloradical **2**.

Table S4. Spin Count obtained from EPR Data during the Transformation 2 + O_2 ⇌ 4 at Variable Temperature

	temperature decreases -------->				temperature increases ----------->		
Time (min) [a]	0	5	24	60	137	171	194
Temperature (°C)	25	15	–25	–120	–25	15	25
Relative spin count (%)[b]	100	99	102	98	95	95	93
Compounds observed	**2**	**2** + **4**	**2** + **4**	**4**	**2** + **4**	**2** + **4**	**2**

[a] Time elapsed since the registration of the first spectrum. [b] Calculated with the quantification tool of Bruker's ESR Studio application (v. 1.90.0). The spin concentrations are given in percent with respect to the spin concentration in the initial solution.

In an additional experiment, a valved EPR tube containing a solution of **2** in acetone was pressurized with dioxygen. The solution was then frozen at 170 K. As expected, complex **2** was completely transformed into **4** under these conditions (Figure S15a). The tube was then brought to room temperature and the dioxygen atmosphere was evacuated and replaced by argon. A new EPR spectrum of the solution was recorded again at 170 K. This spectrum corroborated the complete recovery of the starting complex **2** (Figure S15b).

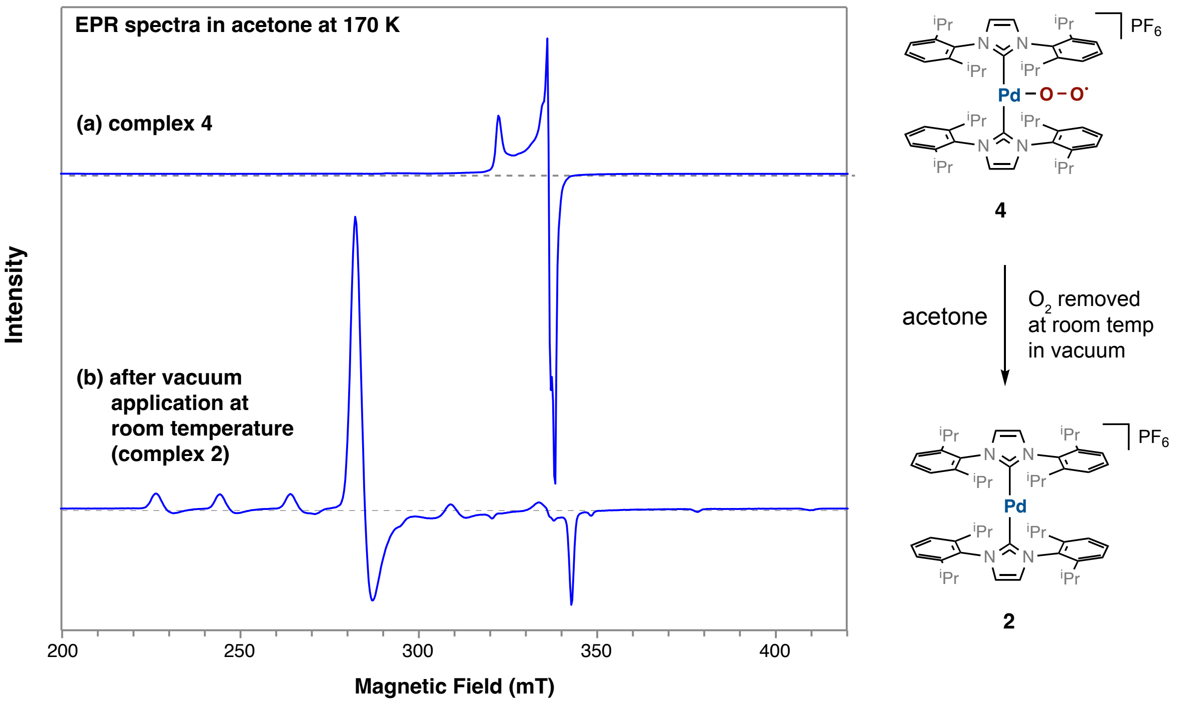

Figure S15. (a) EPR spectrum of complex **4** in frozen acetone; the sample was obtained by cooling a solution of **2** under an atmosphere of O_2. (b) EPR spectrum of the upper sample after vacuum treatment at room temperature showing reversibility towards the formation of complex **2**.

4.4. Reaction of 2 with Dioxygen in 1,2-Difluorobenzene (Figure S16)

EPR study. The behavior described above in acetone is very similar to that observed in 1,2-difluorobenzene, with the only limitation being that this solvent freezes at –35 °C. However, the

transformation of **2** into **4** in the presence of 1 bar oxygen also occurs in this solvent at low temperature, with only a residual amount of **2** being observed when the solution is cooled to 200 K (–73 °C, Figure S16).

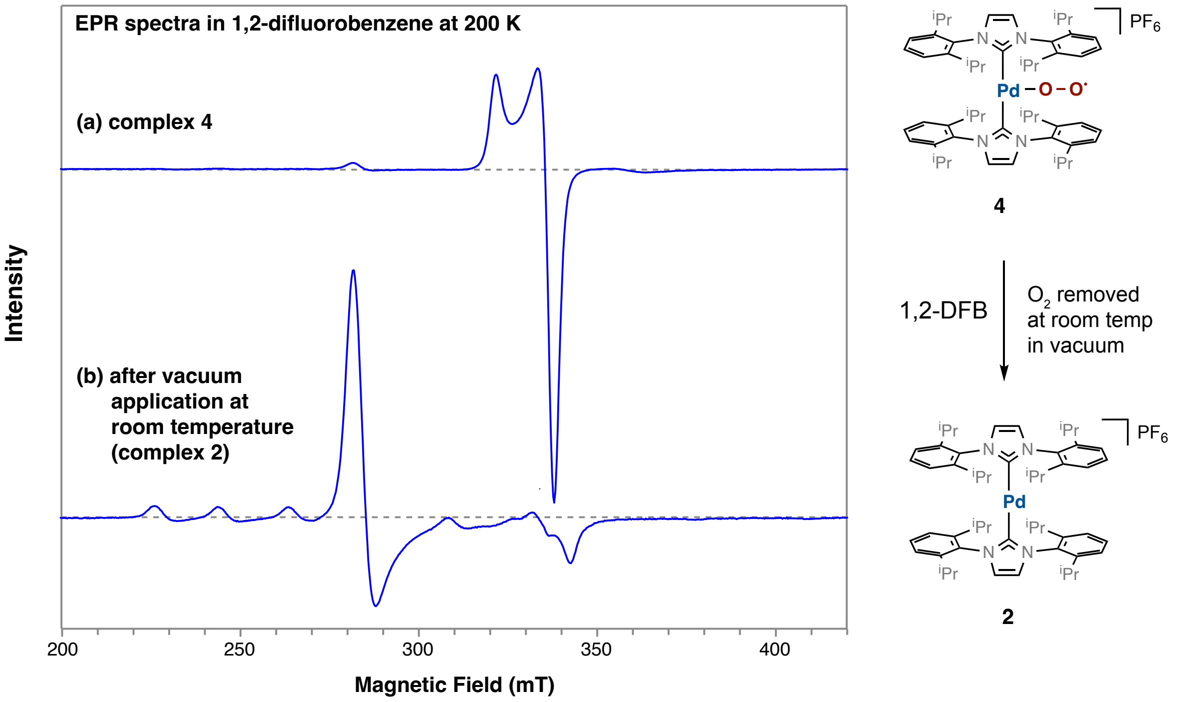

Figure S16. (a) EPR spectrum of complex **4** in frozen 1,2-difluorobenzene; the sample was obtained by cooling a solution of **2** under an atmosphere of O_2. (b) EPR spectrum of the upper sample after vacuum treatment at room temperature showing reversibility towards the formation of complex **2**.

4.5. Reaction of 2 with Dioxygen in Acetonitrile (Figure S17)

EPR study. A valved EPR tube containing a solution of complex **2** in acetone was pressurized with 1 bar of O_2 as described in Section 4.2. EPR spectra were then recorded at 30 °C (Figure S17a) and –100 °C (Figure S17b). The tube was then brought to room temperature and the dioxygen atmosphere was evacuated and replaced by argon. The spectrum of the dissolution was recorded again at –100 °C (Figure S17c). Under these conditions, the recorded EPR spectrum show only the resonances corresponding to **4•NCMe**.

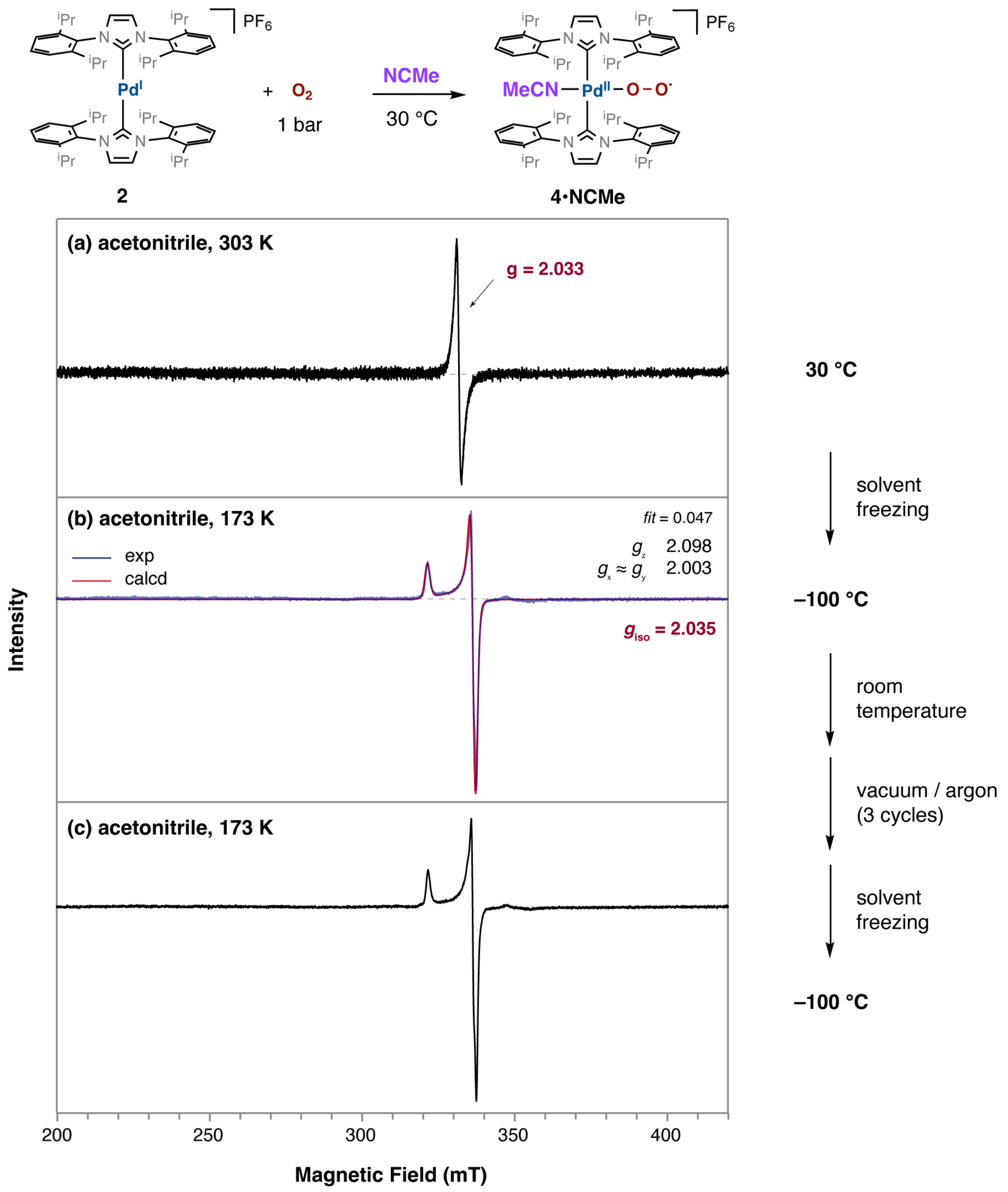

Figure S17. EPR spectra of complex **4•NCMe** in acetonitrile (a) at 30 °C and (b) at −100 °C. (c) EPR spectrum of the same solution at −100 °C after 3 vacuum/argon cycles at room temperature.

4.6. EPR Data and Spectra of 4 in Frozen Solvents (Table S5 and Figure S18)

EPR data for complex **4** in frozen 1,2-difluorobenzene, acetone, and acetonitrile are given in Table S5. Experimental and fitted EPR spectra are shown in Figure S18.

Table S5. EPR Data for Complex 4 in Frozen Solvents

Solvent	Temperature (K)	g_z	g_x	g_y
1,2-Difluorobenzene	200 K	2.099	2.008	2.002
Acetone	170 K	2.099	2.012	2.002
Acetonitrile	133 K	2.098	2.003	2.003

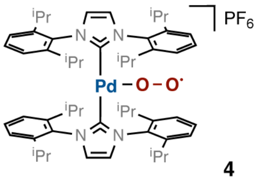

4

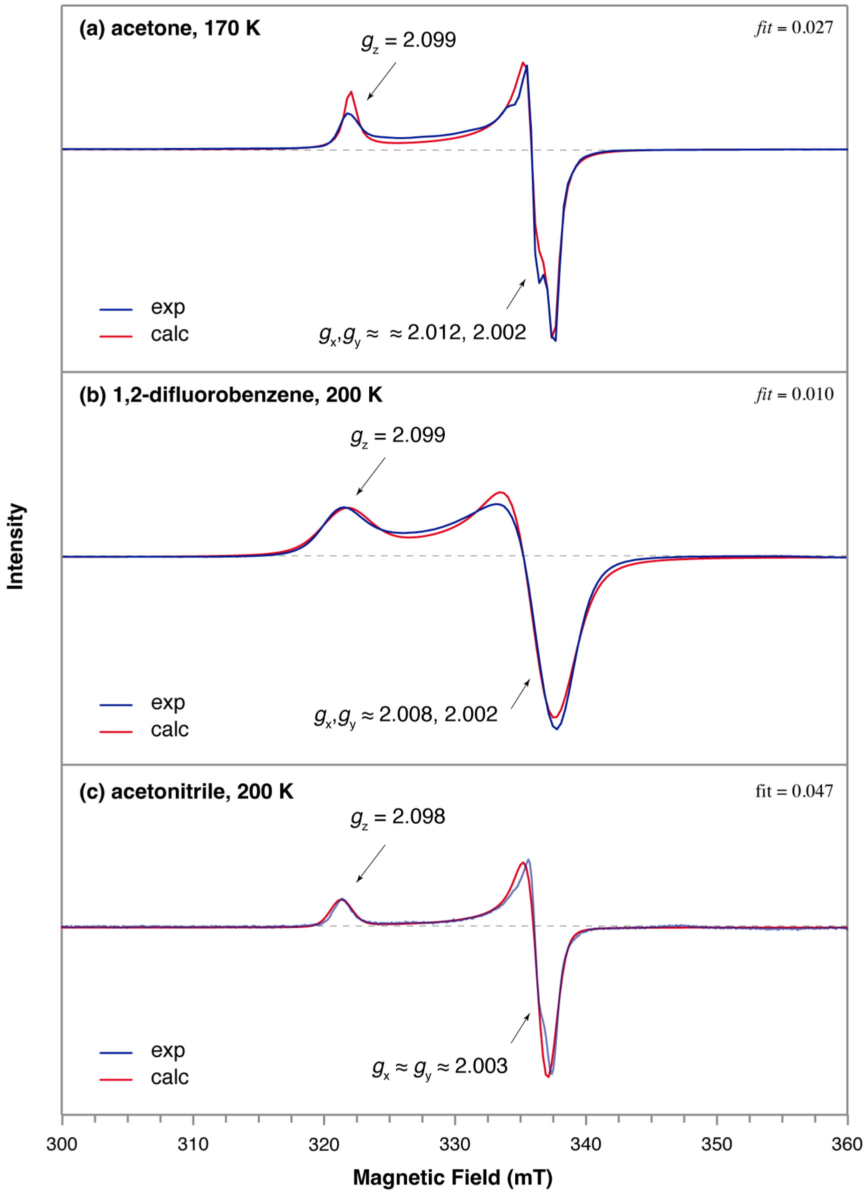

Figure S18. Experimental and fitted EPR spectra of complex **4** (a) in acetone at 170 K, (b) in 1,2-difluorobenzene at 200 K, and (c) in acetonitrile at 200 K. Complex **4** was obtained in situ by pressurizing solutions of **2** in a valved EPR tube with 1 bar of O_2.

5. Cyclic Voltammetry Studies (Figures S19-S21)

Cyclic voltammograms for oxidation of **1** and reduction of **2** are shown in Figure S19. The formal electrode potential ($E_{1/2}$) is −1.020 V relative to Fc^+/Fc for the oxidation of complex **1**. The peak−to-peak potential separation (ΔE_p) is 91 mV at ν = 10 mV s^{-1} which deviates from the value of 57 mV expected for a electrochemically-reversible one-electron oxidation.[15] The Fc^+/Fc redox couple shows a ΔE_p = 108 mV under the same conditions. The formal electrode potential ($E_{1/2}$) is −0.992 V relative to Fc^+/Fc for the reduction of complex **2**. The peak-to-peak potential separation (ΔE_p) is 79 mV at ν = 10 mV s^{-1} and i_p^{ox} / i_p^{red} is 0.96. Randles-Sevcik plots and plots of peak potentials versus the square root of scan rate for complexes **1** and **2** are shown in Figures S20 and S21, respectively.

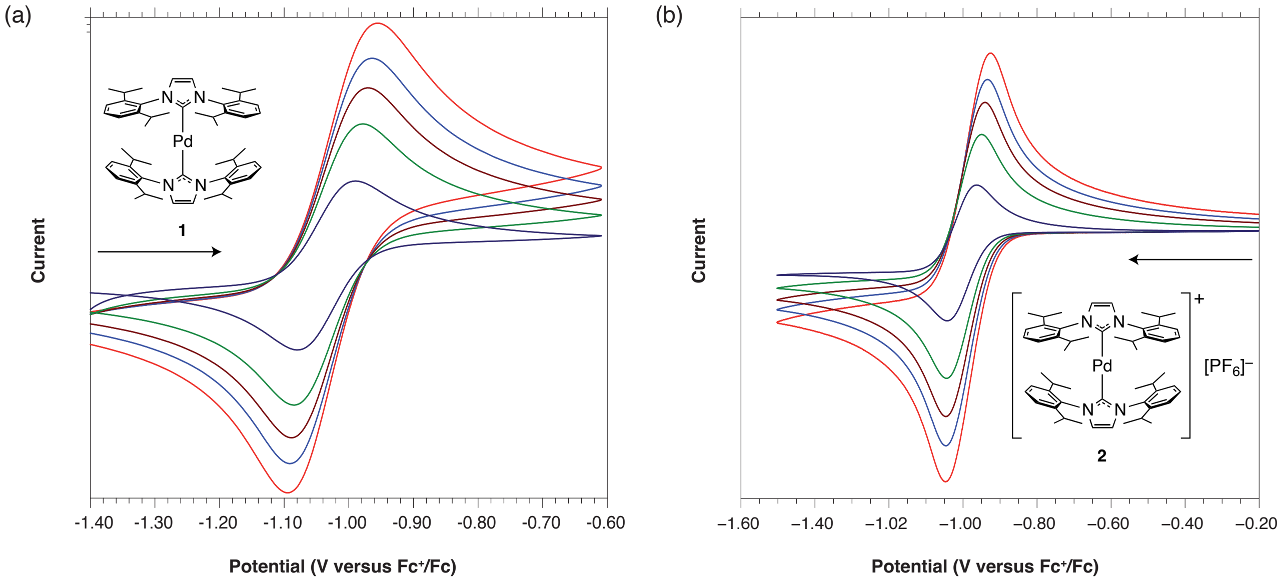

Figure S19. (a) Cyclic voltammogram for the oxidation of **1** in 1,2-difluorobenzene (~ 2 mM **1**, 0.2 M [^{n}Bu$_4$N][PF$_6$] electrolyte; scan rates = 10, 30, 50, 70, 100 mV s^{-1}). (b) Cyclic voltammogram for the reduction of **2** in 1,2-difluorobenzene (~ 2 mM **1**, 0.2 M [^{n}Bu$_4$N][PF$_6$] electrolyte; scan rates = 10, 30, 50, 70, 100 mV s^{-1}).

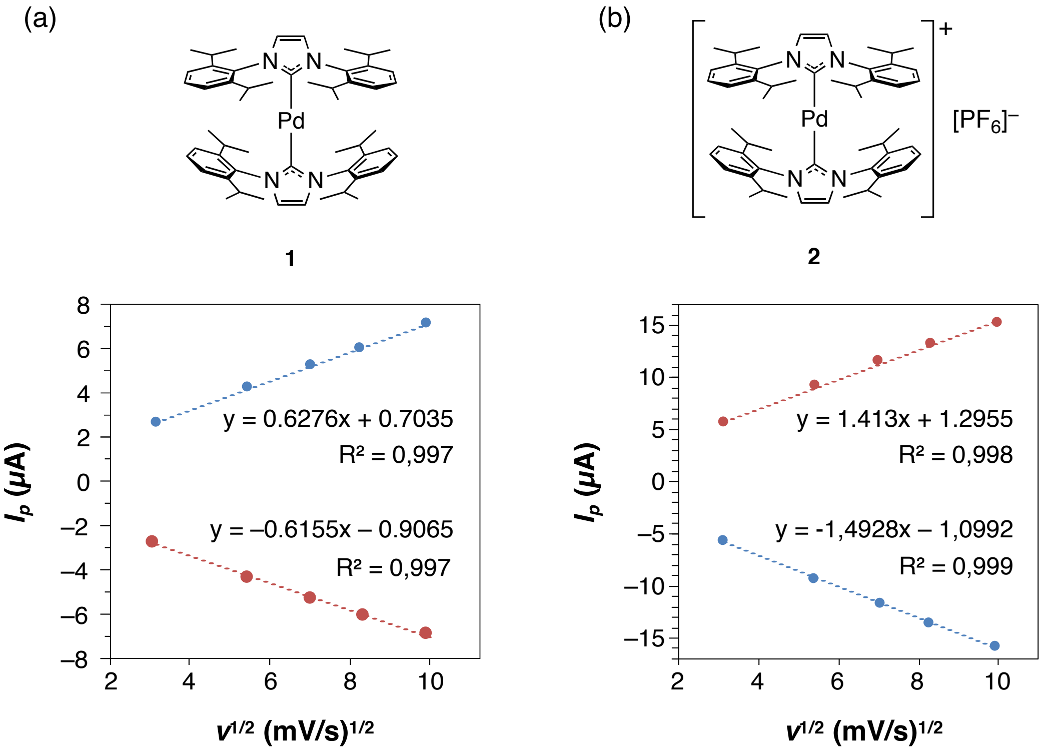

Figure S20. Randles-Sevcik plots of peak current (i_p) versus the square root of scan rate ($\nu^{1/2}$) (a) for complex **1** and (b) for complex **2**.

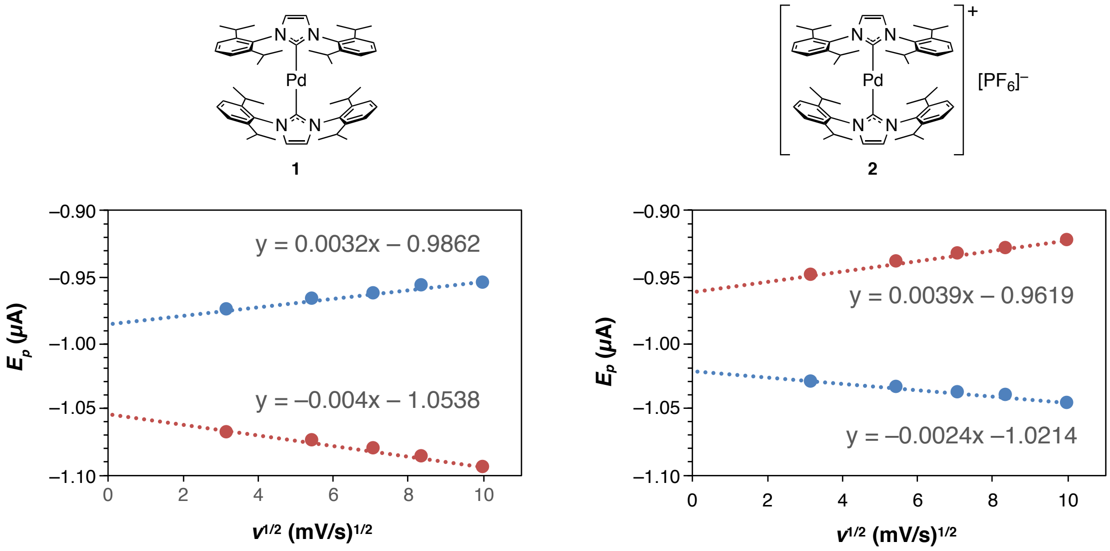

Figure S21. Plots of peak potentials (E_{pa} and E_{pc}) versus the square root of scan rate ($\nu^{1/2}$) for complexes **1** and **2**.

6. Procedure for the Assignment of ^{1}H NMR Resonances

6.1. Complex 2 (Table S6 and Figure S22)

The assignment of resonances for complex **2** in CD_2Cl_2 (Table S6) was made according to the following procedure. First, the iNMR 6.0 for Mac software[16] was used to deconvolute the spectrum and to obtain accurate chemical shifts, linewidths and integral values for all the resonances (Figure S22). The initial assignment of resonances was based on their integral values. In the event of doubt, the widest signal was assigned to the protons closest to the palladium atom in the molecular structure of the compound. As a final check, the experimental longitudinal relaxation times T_1 were compared with the values calculated from the distances of the corresponding protons to the palladium nucleus. The calculation was performed using equation (S1)[17-19] and the aryl proton in para (Ar-*p*-C*H*) as reference. The agreement between calculated and experimental T_1 values (the two columns on the right hand side of Table S6) was acceptable considering the limitations of the model.

$$\frac{T_1}{T_1^{ref}} = \frac{d(\mathrm{H}\cdots\mathrm{Pd})^6}{d(\mathrm{H}^{\mathrm{ref}}\cdots\mathrm{Pd})^6} \tag{S1}$$

Table S6. ^{1}H NMR Parameters for Complex 2 in CD_2Cl_2 at 298 K

Assignment	Number of nuclei	Pd···H (Å)^a	Experimental NMR data				T_1 calcd. (ms)^d
			Chemical shift (δ, ppm)	Measured relative area	$\Delta\nu_{1/2}$ (Hz)^b	T_1 (ms)^c	
Imz-C*H*	4 H	5.17	18.46	4 H	320	4.0 ± 0.5	2.3
Ar-*m*-C*H*	8 H	5.68	8.62	9.4 H	64	5.5 ± 0.3	4.0
Me(1)	24 H	3.93	6.99	20 H^e	723	0.4 ± 0.1	0.4
Ar-*p*-C*H*	4 H	6.24	6.90		41	7.0 ± 0.6	7.0
Me(2)	24 H	6.28	4.12	29 H	96	5.3 ± 0.3	7.3
C*H*Me$_2$	8 H	4.27	0.98	10 H	485	0.6 ± 0.1	0.7

^{a} Crystallographic distance determined by X-ray diffraction. In the case of equivalent protons, the arithmetic average distance is given. ^{b} Line width at half-maximum. ^{c} T_1 values were obtained at 500 MHz. ^{d} T_1 values calculated from the measured Pd···H distances, according to equation (1).[17-19] The aryl proton in para (Ar-*p*-CH) was used as reference. ^{e}Me(1) and Ar-*p*-C*H* resonances overlap.

As a further check, the variable-temperature ^{1}H NMR spectra of complex **2** were recorded in CD_2Cl_2 between −60 and 30 °C (Figure S8). ^{1}H NMR chemical shifts, linewidths and integral values at each temperature (see Table S2) were obtained by deconvolution of the NMR spectra as explained above.[16] The chemical shift values were plotted against $1/T$ to obtain the corresponding Curie plot (Figure 4). The convergence of the chemical shift values to the expected diamagnetic regions at $1/T = 0$ was used as a check on the assignment.

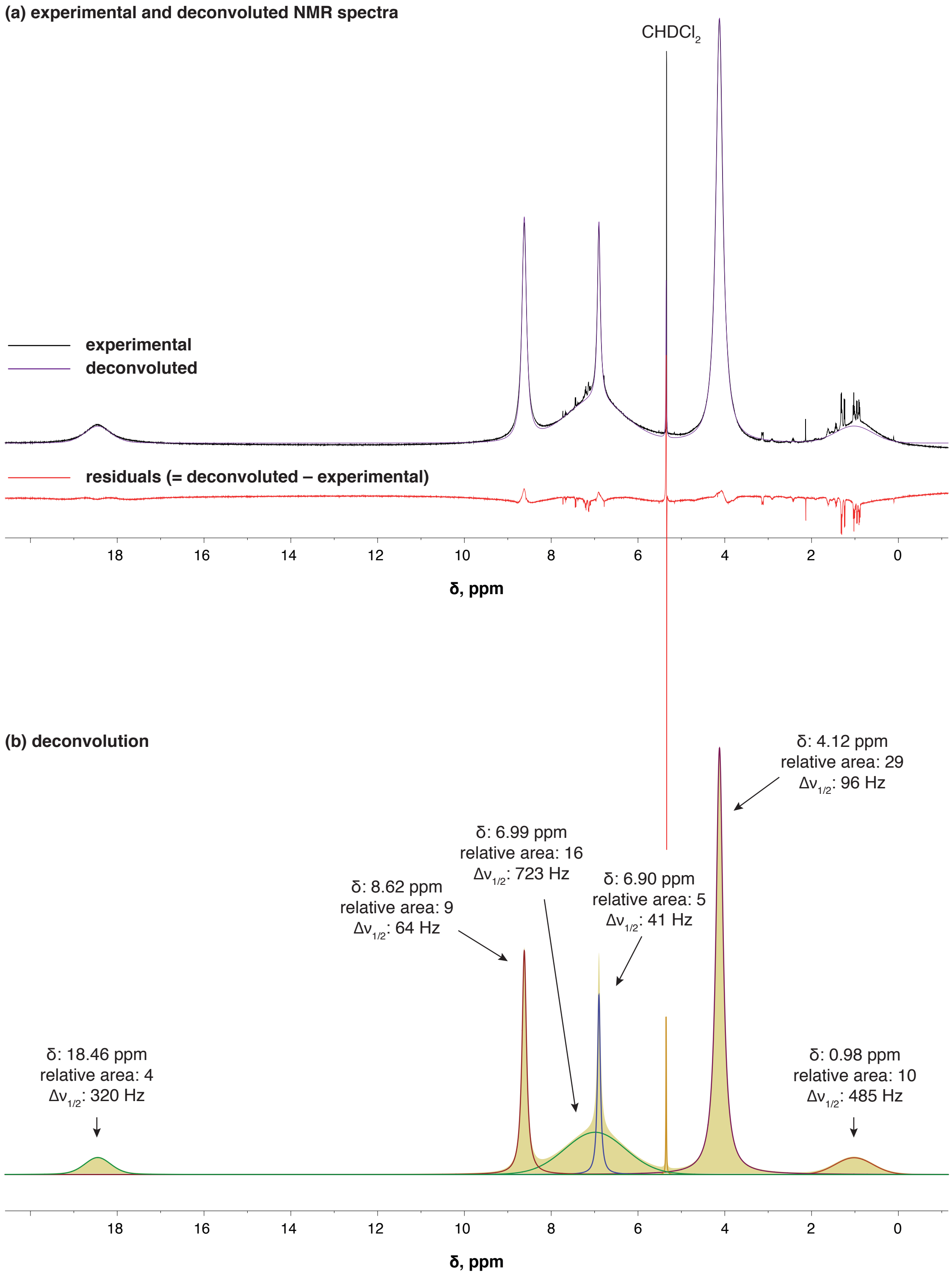

Figure S22. (a) Experimental and deconvoluted ^{1}H NMR spectrum (300 MHz) for **2** in CD_2Cl_2 at 298.3 K. (b) Deconvoluted resonances.

6.2. Complex 4 (Figure S23)

Resonances of complex **4** in acetone-d_6 were assigned as follows. First, the ^{1}H NMR spectrum of complex **2** was recorded in acetone-d_6 at −60 °C using a valved NMR tube (Figure S23a). The tube was then pressurized with O_2 (1 bar) to form the equilibrium mixture of **2** and **4** and the ^{1}H NMR

spectrum was recorded again at the same temperature (Figure S23b). Then, the first spectrum was subtracted from the second spectrum to uncover the resonances of complex **4** (Figure S23c). The intensities of the resonances of complex **2** in the first spectrum were previously adjusted to match as much as possible the corresponding resonances in the second spectrum. Finally, the iNMR 6.0 for Mac software[16] was used to deconvolute the spectrum and to obtain chemical shifts, linewidths and integral values (Figure S23d).

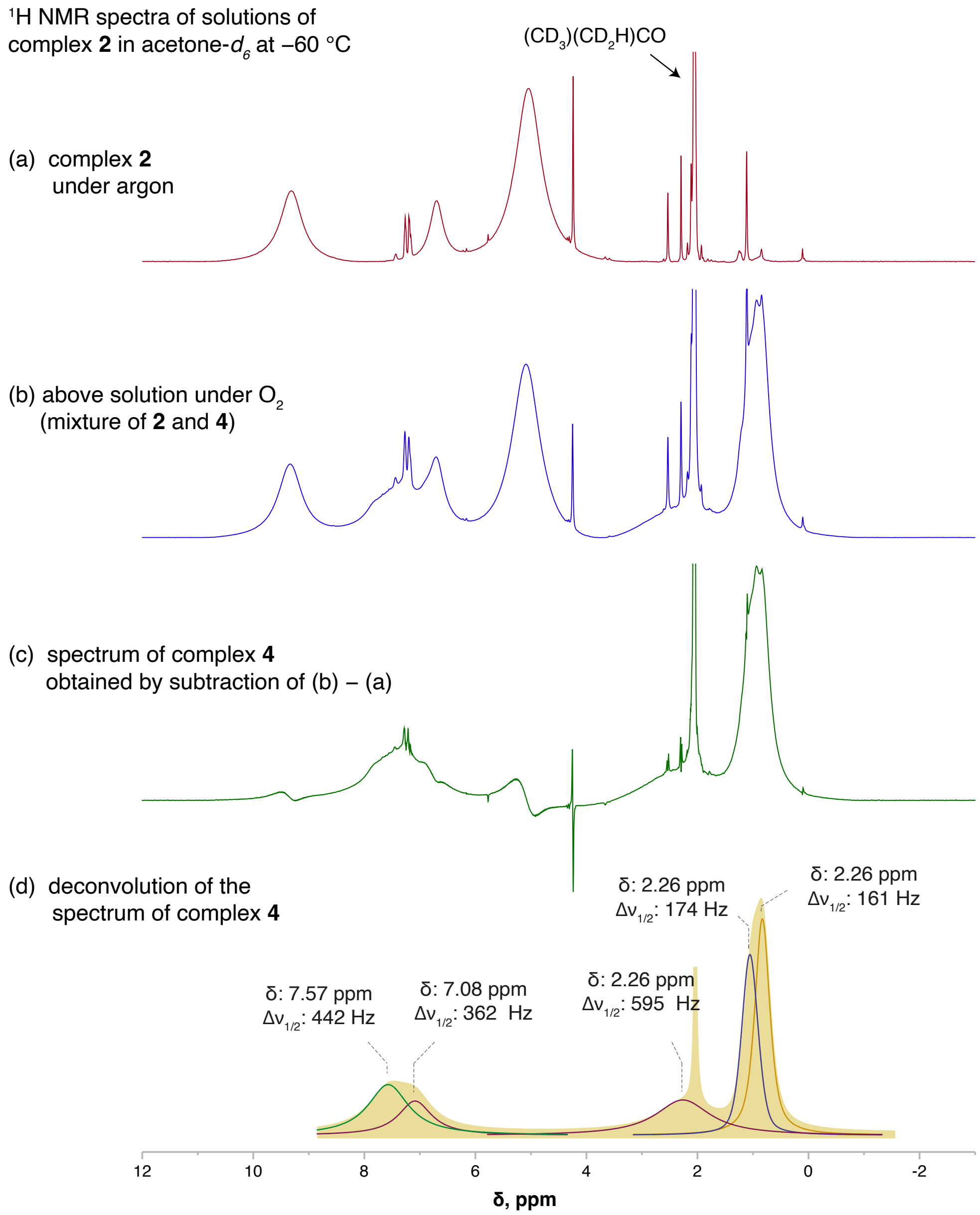

Figure S23. (a) ^{1}H NMR spectrum of complex **2** in acetone-d_6 at −60 °C. (b) ^{1}H NMR spectrum of the mixture of **2** and **4** formed upon pressurizing with O_2 (1 bar) at the same temperature. (c) The resonances of complex **4** are revealed by subtraction of spectrum b minus spectrum a. (d) Deconvolution of the resonances of complex **4**.

7. Procedure for the Magnetic Susceptibility Measurements (Figure S24)

The magnetic susceptibility of complex **2** was measured in solution employing the Evans method.[20,21] A 2-mm glass capillary was filled with a solution of **2** (0.010 g, 9.722 × 10^{-3} mol/L) in 0.9 mL of CD_2Cl_2 and 0.1 mL of cyclohexane. The glass capillary was inserted inside an NMR tube filled with 0.45 mL of CD_2Cl_2 and 0.05 mL of cyclohexane. The ^{1}H NMR resonance of the cyclohexane inside the capillary was shifted 25.5 Hz in respect to the outer cyclohexane resonance in a 500-MHz NMR spectrometer at 298 K (Figure S24).

Mass and molar magnetic susceptibilities (χ_{mass} and χ_{mol}) of **2** were calculated making use of equations (S2) and (S3):[22,23]

$$\chi_{\text{mass}} = \chi_0 + \frac{3000\,\Delta\nu}{4\pi\,\nu_0\,c\,M} = 6.48 \times 10^{-7}\ \text{cm}^3/\text{g} \qquad \text{(S2)}$$

$$\chi_{\text{mol}} = \chi_{\text{mass}} \times M = 6.67\ \times 10^{-4}\ \text{cm}^3/\text{mol} \qquad \text{(S3)}$$

where χ_0 is the diamagnetic correction due to solvent (–0.543 × 10^{-6} cm^3/g for CD_2Cl_2[24] and –0.809 × 10^{-6} cm^3/mol for cyclohexane),[25] $\Delta\nu$ is the observed frequency shift of the cyclohexane reference resonance (25.5 Hz), ν_0 is the frequency of the nucleus under observation (500 10^6 Hz), *c* is the molar concentration of the substance (9.722 × 10^{-3} mol/L), and *M* is the molar mass (1028.56 g/mol).

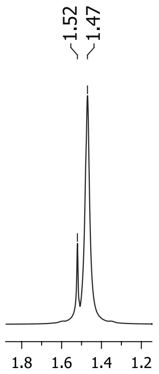

Figure S24. Determination of the magnetic susceptibility of **2** in CD_2Cl_2 by the Evans method. ^{1}H resonances for cyclohexane in the inner and outer tubes.

The diamagnetic susceptibility of the complex was estimated by means of eq. (4) (*MW* is the molecular weight)[25] and was subtracted from the total susceptibility to determine the paramagnetic contribution (χ_P, eq. (S5)).

$$\chi_D = -\frac{MW}{2} \times 10^{-6} = -5.14 \times 10^{-4}\ \text{cm}^3/\text{mol} \qquad \text{(S4)}$$

$$\chi_P = \chi_{\text{mol}} - \chi_D = 1.18 \times 10^{-3}\ \text{cm}^3/\text{mol} \qquad \text{(S5)}$$

The effective magnetic moment (μ_{eff}), in Bohr magnetons, was determined using eq. (S6).

$$\mu_{eff} = 2.828\mu_B\sqrt{\chi_P \times T} = 1.68\ \mu_B \qquad \text{(S6)}$$

8. Crystallographic Data

8.1. Selected Crystallographic Data for Complex 2 (Table S7 and Figures S25 and S26)

Table S7. Selected Crystallographic Data for the Pd(I) Complex [Pd^{I}(IPr)$_2$]PF$_6$ (2) and Comparison with Data Reported for the Pd(0) Complex [Pd^{0}(IPr)$_2$] (1)

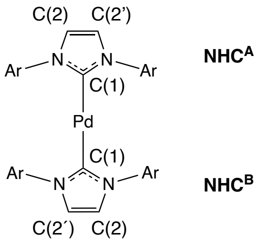

Parameter	[Pd^{I}(IPr)$_2$]PF$_6$ (**2**)			[Pd^{0}(IPr)$_2$] (**1**)[26,27]	Difference
	NHC^{A}	NHC^{B}	Average	Average	
Pd-C(1)	2.091(6)	2.021(7)	2.056±0.038	2.013±0.014	0.043 Å
C(1)-N	1.359(5)	1.399(6)	1.379±0.021	1.367±0.019	0.012 Å
N-C(2)	1.382(6)	1.392(6)	1.387±0.006	1.377±0.017	0.010 Å
C(2)-C(2')	1.334(10)	1.340(10)	1.337±0	1.330±0.015	0.007 Å
N-Ar	1.450(6)	1.443(6)	1.4465±0.003	1.439±0.012	0.0075 Å
C(1)-Pd-C(1)	180	180	180.0±0	177.2±1.3	2.8°
N-C(1)-N	103.2(5)	99.5(6)	101.35±2.05	101.303±0.293	0.05°
IPr-IPr dihedral			39.4°	46.8°	7.4°

(a)

(b)

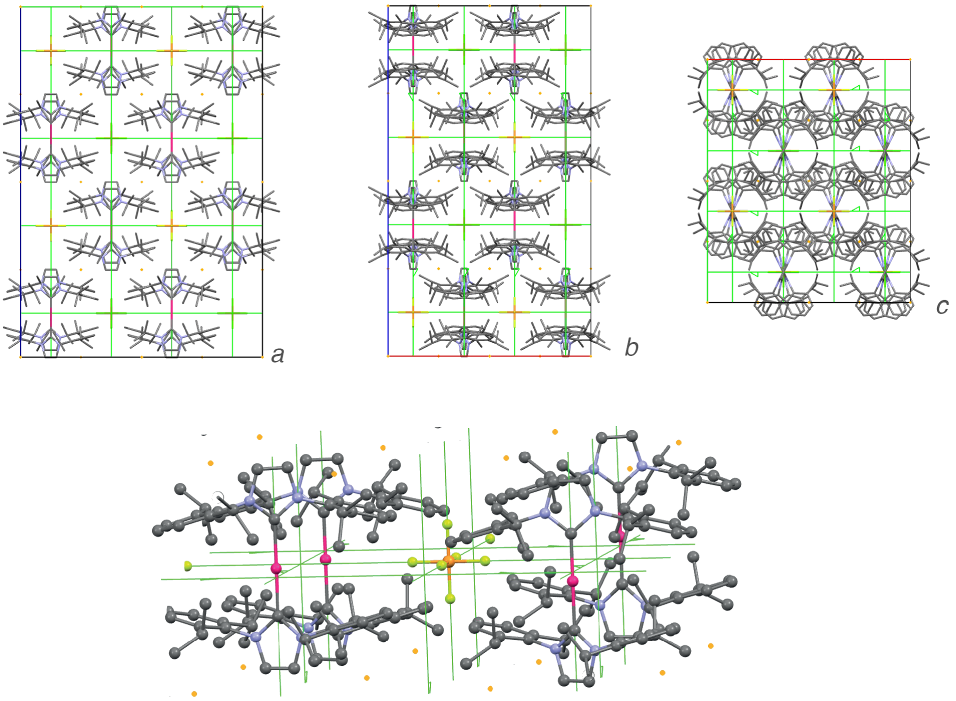

Figure S25. (a) View of the crystal packing of **2** along crystallographic axes *a*, *b*, and *c*. Green lines represent two-fold symmetry axes and the orange points are inversion centers. (b) Detail of the crystal packing of compound **2**.

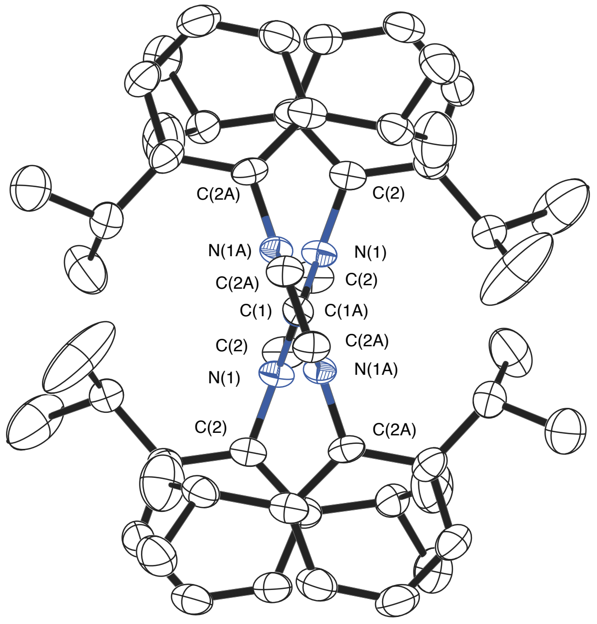

Figure S26. ORTEP view along the C(1)-Pd-C(1A) direction of the cationic $[Pd(IPr)_2]^+$ unit in compound **2**. This direction corresponds with a crystallographic 2-fold rotation axis.

8.2. Crystal Structure Determination Details (Table S8)

Suitable single crystals of **2** were obtained by overnight cooling at 5 °C of a concentrated solution of the complex (50 mg) in 1,2-difluorobenzene (5 mL). A summary of crystal data, data collection, and refinement parameters for the structural analysis is given in Table S8. Crystals were glued to a glass fiber using an inert polyfluorinated oil and mounted in the low temperature N_2 stream of a Bruker-Nonius Kappa-CCD diffractometer equipped with an area detector and an Oxford Cryostream 700 unit.

Intensities were collected using graphite-monochromated Mo-Kα radiation (λ = 0.71073 Å). Data were measured with exposure times of 19 s per frame (2 sets; 109 frames; phi/omega scans; 1.9° scan-width). Raw data were corrected for Lorenz and polarization effects. The structure was solved by direct methods, completed by subsequent difference Fourier techniques and refined by full-matrix least squares on F^2 (SHELXL-97).[28] Anisotropic thermal parameters were used in the last cycles of refinement for the non-hydrogen atoms. Absorption correction procedures were carried out using the multiscan SORTAV (semiempirical from equivalent) program. Hydrogen atoms were included in the last cycle of refinement from geometrical calculations and refined using a riding model. All the calculations were made using the WINGX system.[29]

Table S8. Crystallographic Data for Compound 2

Empirical formula	$C_{54}H_{72}F_6N_4PPd$
Formula weight	1028.52
Crystal size (mm)	0.48 × 0.38 × 0.31
Color / habit	yellow / prism
Temperature (K)	200(2)
Wavelength (Å)	0.71073
Crystal system	orthorhombic
Space group	F d d d
a (Å)	21.4684(16)
b (Å)	25.604(2)
c (Å)	37.010(4)
α (deg)	90
β(deg)	90
γ (deg)	90
Volume ($Å^3$)	20343(3)
Z	16
Calculated density (g/cm^3)	1.343
μ (mm^{-1})	0.458
F(000)	8624
θ range (deg)	3.006 to 27.499
Limiting indices (*h*, *k*, *l*)	-27 ≤ h ≤ 27, -31 ≤ k ≤ 32, -48 ≤ l ≤ 42
Number of reflns collected	33147
Number of reflns unique / r_{int}	5763 / 0.1067
Number of reflns observed [$i > 2\sigma(i)$]	3553
Completeness to θ	99.6%
Absorption correction	multi-scan
Max. and min. transmission	0.834 and 0.890
Refinement method	full-matrix least-squares on F^2
Number of data / restraints / parameters	5763 / 6 / 312
Goodness of fit on F^2	1.104
R_1 / wR_2 [$I > 2\sigma(I)$] [a]	0.0594 / 0.1261
R_1 / wR_2 (all data)	0.1277 / 0.1678
Largest diff. peak and hole (e/$Å^3$)	1.44 and –1.08

[a] $R_1 = \Sigma||F_o|-|F_c|| / \Sigma|F_o|$; $wR_2 = [\Sigma w(F_o^2-F_c^2)/\Sigma w(F_o^2)^2]^{1/2}$.

9. Computational Details

9.1. Mulliken Atomic Spin Densities for Complexes 2 and 4 (Tables S9 and S10)

Table S9. Mulliken Atomic Spin Densities Calculated for Complex 2 by DFT Methods (B3LYP/LanL2DZ)[a,b]

Atom	Spin density	Atom	Spin density	Atom	Spin density
Pd	0.886652	N	−0.002443	N	−0.002457
C(NHC[A])	0.055595	N	−0.002448	Other C and H	from + 0.001351 to −0,001616
C(NCH[B])	0.055685	N	−0.002452		

[a] See Section 9.2 for computational details. [b] ⟨S^2⟩ = 0.7500.

Table S10. Mulliken Atomic Spin Densities Calculated for Complex 4 by DFT Methods (B3LYP/LanL2DZ)[a]

Atom	Spin density	Atom	Spin density	Atom	Spin density
Pd	−0.504542	C(NHC[A])	-0.055895	Other C, N and H	from + 0.002538 to −0.001228
O(α)	0.756997	C(NCH[B])	-0.054076		
O(β)	0.854569				

[a] See Section 9.2 for computational details. [b] ⟨S^2⟩ = 0.7618.

9.2. Computational Details (Table S11)

Calculations were performed at the density functional theory (DFT) level of theory with the Gaussian 09 Rev. D.01 software package[30] and using the B3LYP functional[31,32] along with the LanL2DZ basis set.[33-35] The molecular structures were optimized in gas phase without any symmetry constraint with very tight convergence criteria (optimized coordinates in Table S11). The molecular structure of compound **2** obtained from X-ray diffraction analysis was used as an initial structural guess for geometry optimization. Frequency calculations were performed at the same level of theory for freely relaxed molecules to distinguish optimized geometries as stationary points on the potential energy surface, which do not exhibit imaginary frequencies. In addition, the stability of the wave functions generated by the self-consistent field (SCF) calculations was checked using the keyword *stable*. Spin contamination was checked. Values of ⟨S^2⟩ deviated less than 5% from the expected value of 0.75 for a system of S = ½ (values in footnote b of Tables S8 and S9).

Table S11. DFT-optimized coordinates (B3LYP/LanL2DZ).

Complex **2**				Complex **3**			
C	−0.005777	2.091145	0.017581	C	−4.257048	3.356431	1.392336
C	−0.662011	4.293248	−0.163657	C	−3.929762	3.307942	0.028752
C	0.641450	4.291325	0.248823	C	−2.401119	4.685089	−2.441215
C	−2.388369	2.573390	−0.721749	C	0.604675	2.034737	−0.047216
C	2.374284	2.565278	0.768727	C	0.527177	4.331960	0.054091
C	−2.642426	2.403070	−2.107524	C	1.799042	3.982800	−0.304487

C	2.625707	2.357273	2.149834	C	−0.594163	−1.997328	0.106495
C	−3.971532	2.133883	−2.496283	C	−0.491595	−4.280079	0.375026
C	3.954703	2.081457	2.534287	C	−1.769647	−3.971040	0.003197
C	−4.999679	2.044496	−1.545709	C	−2.252254	3.239824	−1.888994
C	4.985262	2.021152	1.584082	C	−1.590729	3.135805	0.612189
C	−4.720875	2.227255	−0.182773	C	−1.889760	3.194022	1.998326
C	4.709138	2.241355	0.226169	C	−3.247436	3.302132	2.365341
C	−3.410664	2.502853	0.260955	C	−2.588323	3.203864	−0.394477
C	3.399267	2.525794	−0.212938	C	−0.808540	3.190147	3.085320
C	−3.149127	2.786019	1.744288	C	−0.767365	4.550613	3.832088
C	3.141095	2.850901	−1.688353	C	−1.001207	2.022769	4.086892
C	−3.794190	1.730999	2.675727	C	1.612233	−3.023567	0.853221
C	3.797238	1.829110	−2.648639	C	2.624475	−3.217778	−0.122940
C	−3.645311	4.210881	2.118523	C	3.958825	−3.274289	0.330730
C	3.628185	4.289918	−2.017757	C	4.266677	−3.160730	1.695018
C	−1.555034	2.560813	−3.175573	C	3.243712	−2.986374	2.639032
C	1.535666	2.482479	3.219558	C	1.892117	−2.917105	2.241118
C	−1.509286	1.355962	−4.148482	C	0.796500	−2.789486	3.305624
C	1.489661	1.250111	4.157169	C	0.996050	−1.537269	4.197063
C	−1.748294	3.887626	−3.960304	C	0.720593	−4.073398	4.175627
C	1.725449	3.786775	4.041961	C	2.307534	−3.444389	−1.604992
C	1.793033	−3.742682	−4.114009	C	3.068709	1.865837	−0.685398
C	−1.766814	−3.871495	4.020604	C	3.403674	1.647839	−2.048723
C	1.421090	−1.217555	−4.090011	C	4.643015	1.030203	−2.318603
C	−1.424880	−1.343820	4.092069	C	5.510797	0.658945	−1.280156
C	3.159302	−2.831121	1.683739	C	5.164332	0.910392	0.055368
C	−3.141438	−2.795825	−1.738309	C	3.940205	1.529585	0.384248
C	3.648331	−4.269586	2.013043	C	3.640172	1.900544	1.841457
C	−3.628319	−4.224505	−2.110516	C	4.407131	3.195631	2.232971
C	3.818186	−1.808469	2.640992	C	3.973275	0.763383	2.836278
C	−3.799295	−1.745951	−2.666348	C	2.519493	2.131218	−3.205588
C	0.005582	−2.104987	−0.019676	C	−3.075993	−1.899853	−0.486600
C	0.663554	−4.302087	−0.248630	C	−3.499611	−1.851048	−1.841278
C	−0.640491	−4.309900	0.162575	C	−4.754828	−1.262418	−2.106052
C	2.385732	−2.565336	−0.772972	C	−5.553333	−0.757636	−1.069829
C	−2.371542	−2.594584	0.725302	C	−3.882719	−1.434965	0.587327
C	3.411866	−2.508388	0.206817	C	−2.699886	−2.479228	−2.988958
C	−3.397295	−2.516214	−0.253305	C	−2.658961	−1.590554	−4.257287
C	4.716921	−2.209171	−0.236348	C	−3.281376	−3.876713	−3.348063
C	−4.704432	−2.237115	0.196893	C	−3.114381	2.252814	−2.712672
C	4.987426	−1.990583	−1.595919	C	−3.849334	−0.450901	2.965858
C	−4.976930	−2.057786	1.561775	C	3.245275	−2.650669	−2.546230
C	3.956145	−2.068701	−2.543807	C	2.367932	−4.961138	−1.941935
C	−3.945633	−2.155576	2.507894	C	2.481961	1.146763	−4.400091
C	2.631863	−2.361543	−2.155219	C	2.995916	3.527655	−3.700068
C	−2.619449	−2.429966	2.112535	C	−3.499723	−1.651844	2.056389
C	1.541137	−2.496183	−3.222827	C	−5.123647	−0.849390	0.262589
C	−1.528588	−2.589769	3.176540	C	−4.179394	−2.943055	2.595875
H	−1.337209	5.106277	−0.370548	H	0.076204	5.296028	0.217682
H	1.312841	5.102379	0.474921	H	2.666470	4.586816	−0.508473
H	−4.205268	2.003088	−3.549152	H	−2.631318	−4.597824	−0.149542
H	4.186448	1.923109	3.583846	H	−0.030872	−5.224860	0.608151
H	−6.018111	1.842768	−1.867907	H	−2.137220	4.714922	−3.506035
H	6.003511	1.813297	1.902955	H	−1.751047	5.392417	−1.910714
H	−5.528616	2.165107	0.540600	H	−3.435008	5.038855	−2.339355
H	5.518760	2.201844	−0.496635	H	−1.205532	2.941521	−2.021091
H	−2.066513	2.757415	1.918608	H	0.169481	3.050743	2.609337
H	2.059407	2.819925	−1.867936	H	−0.587489	5.383857	3.141111

H	−0.579704	2.613418	−2.677456	H	0.034738	4.550328	4.581121
H	0.561407	2.548069	2.721014	H	−1.712624	4.747462	4.353102
H	−3.485330	0.714485	2.408764	H	−1.947385	2.114598	4.634492
H	3.495643	0.802904	−2.412401	H	−0.188969	2.020662	4.825017
H	−3.499802	1.921886	3.715979	H	−1.002331	1.052861	3.573960
H	3.504015	2.048103	−3.683661	H	−0.171869	−2.678461	2.803711
H	−4.890034	1.771412	2.635017	H	1.926412	−1.598148	4.775179
H	4.892611	1.877012	−2.603695	H	0.167911	−1.445621	4.911498
H	1.838470	−4.661662	−3.515990	H	1.034041	−0.620242	3.595574
H	−1.800568	−4.768430	3.389288	H	−0.089544	−3.989425	4.911246
H	0.986835	−3.855162	−4.850250	H	1.656718	−4.237086	4.723980
H	−0.960436	−4.001457	4.753764	H	0.532686	−4.963905	3.562766
H	2.738958	−3.654544	−4.663190	H	1.286177	−3.099061	−1.801014
H	−2.714602	−3.814475	4.570774	H	2.567943	2.112018	1.936354
H	−4.727854	4.301494	1.962601	H	5.491166	3.044581	2.153317
H	4.709378	4.383657	−1.854511	H	4.179675	3.475511	3.269605
H	−3.436776	4.422526	3.175000	H	4.138070	4.040308	1.587087
H	3.422310	4.531789	−3.068258	H	3.492657	−0.177392	2.548716
H	−3.154595	4.984061	1.514389	H	3.630473	1.036171	3.842848
H	3.129285	5.040985	−1.392802	H	5.053971	0.584761	2.899944
H	2.338820	−1.030758	−4.661646	H	1.492133	2.240238	−2.835604
H	−2.345128	−1.189976	4.669386	H	−1.665363	−2.625625	−2.657155
H	0.598911	−1.323692	−4.809160	H	−2.311165	−0.576917	−4.032072
H	−0.602265	−1.468101	4.807851	H	−3.645331	−1.516903	−4.732103
H	1.222698	−0.334231	−3.470158	H	−1.974354	−2.028217	−4.994221
H	−1.236041	−0.435012	3.507291	H	−4.322351	−3.790642	−3.684793
H	2.078129	−2.800103	1.866996	H	−3.262288	−4.562899	−2.492605
H	−2.059915	−2.758254	−1.918142	H	−2.699528	−4.332395	−4.159262
H	−2.430484	1.277777	−4.739192	H	−4.173682	2.539395	−2.711423
H	2.411041	1.154268	4.744931	H	−3.039967	1.233096	−2.319796
H	−0.675845	1.472237	−4.852975	H	−2.777937	2.246767	−3.757362
H	0.656470	1.346277	4.864961	H	−3.444638	−0.617231	3.972602
H	−1.372005	0.411371	−3.608196	H	−4.933999	−0.324739	3.072007
H	1.351643	0.321625	3.589703	H	−3.435615	0.485014	2.576613
H	4.729000	−4.362543	1.845848	H	4.275050	−3.028438	−2.507226
H	−4.709308	−4.323339	−1.948782	H	3.264773	−1.585700	−2.290824
H	3.446516	−4.510361	3.064590	H	2.900170	−2.750278	−3.583026
H	−3.423785	−4.434161	−3.168173	H	1.660021	−5.542137	−1.337853
H	3.147702	−5.021751	1.390858	H	3.373245	−5.361917	−1.759320
H	−3.128442	−4.994067	−1.509325	H	2.124495	−5.125226	−2.999313
H	−2.707203	3.895056	−4.493942	H	1.735164	1.481298	−5.131046
H	2.683183	3.780466	4.577729	H	2.216889	0.132138	−4.085243
H	−1.731008	4.757496	−3.291690	H	3.446917	1.104048	−4.920681
H	1.708459	4.675007	3.397918	H	4.026762	3.474694	−4.072818
H	−0.949115	4.012980	−4.702108	H	2.964557	4.280135	−2.903597
H	0.924488	3.890205	4.785219	H	2.356597	3.877828	−4.520399
H	3.516935	−0.782303	2.404080	H	−2.414309	−1.798868	2.116984
H	−3.498362	−0.726916	−2.399874	H	−5.272218	−2.847585	2.562423
H	3.526981	−2.025805	3.676932	H	−3.886786	−3.121990	3.638547
H	−3.507177	−1.933520	−3.707841	H	−3.901342	−3.826847	2.009364
H	4.913436	−1.857255	2.594249	H	−5.296709	3.447241	1.696512
H	−4.894573	−1.795934	−2.622001	H	−4.721330	3.363288	−0.712706
H	1.338802	−5.110007	−0.474602	H	−3.515566	3.355055	3.417166
H	−1.311692	−5.125932	0.370784	H	4.760687	−3.419606	−0.386781
H	5.527542	−2.155959	0.484401	H	5.301373	−3.218732	2.023248
H	−5.514947	−2.168770	−0.522742	H	3.495873	−2.915762	3.693709
H	6.002203	−1.770124	−1.917375	H	4.934802	0.843840	−3.347458
H	−5.993154	−1.852321	1.888542	H	6.461968	0.187224	−1.513559

H	4.183352	−1.911886	−3.594806	H	5.854756	0.635295	0.847090
H	−4.174315	−2.028278	3.562487	H	−5.111951	−1.204606	−3.129780
H	0.576261	−2.641770	−2.723142	H	−6.517487	−0.310487	−1.298400
H	−0.561257	−2.704677	2.673520	H	−5.764453	−0.477088	1.055969
N	−1.045978	2.948870	−0.299423	N	−0.191324	3.135859	0.207416
N	1.031093	2.945837	0.353634	N	1.835871	2.580071	−0.368597
N	1.046648	−2.954862	−0.354098	N	−1.823627	−2.576818	−0.160778
N	−1.030616	−2.967274	0.297082	N	0.216374	−3.069218	0.434110
Pd	−0.000361	−0.006962	−0.001540	O	−0.231257	0.078580	−2.185262
				O	0.262870	−0.893925	−2.894515
				Pd	0.000400	0.018807	−0.011004

10. References

(1) Walter, R.; Meyer, H.; Voss, S. Palladium(0)-dibenzylidene acetone complexes, US Patent 2010/0167408A1, Jul 1, 2010.

(2) Kapdi, A. R.; Whitwood, A. C.; Williamson, D. C.; Lynam, J. M.; Burns, M. J.; Williams, T. J.; Reay, A. J.; Holmes, J.; Fairlamb, I. J. S. The Elusive Structure of $Pd_2(dba)_3$. Examination by Isotopic Labeling, NMR Spectroscopy, and X-ray Diffraction Analysis: Synthesis and Characterization of Pd_2(dba-Z)$_3$ Complexes. *J. Am. Chem. Soc.* **2013**, *135*, 8388-8399.

(3) Huang, J.; Nolan, S. P. Efficient Cross-Coupling of Aryl Chlorides with Aryl Grignard Reagents (Kumada Reaction) Mediated by a Palladium/Imidazolium Chloride System. *J. Am. Chem. Soc.* **1999**, *121*, 9889-9890.

(4) Jafarpour, L.; Stevens, E. D.; Nolan, S. P. A sterically demanding nucleophilic carbene: 1,3-bis(2,6-diisopropylphenyl)imidazol-2-ylidene). Thermochemistry and catalytic application in olefin metathesis. *J. Organomet. Chem.* **2000**, *606*, 49-54.

(5) Connelly, N. G.; Geiger, W. E. Chemical Redox Agents for Organometallic Chemistry. *Chem. Rev.* **1996**, *96*, 877-910.

(6) Tait, C.; Lehner, J.; Nehrkorn, J.; Krzyaniak, M.; Ho, A.; Stoll, S. *EasySpin, software package for spectral simulation and analysis in EPR*, version 5.2.25, 2014, http://www.easyspin.org (accessed 11/18/2023).

(7) Stoll, S.; Schweiger, A. EasySpin, a comprehensive software package for spectral simulation and analysis in EPR. *J. Magn. Reson.* **2006**, *178*, 42-55.

(8) Etienne, E.; Le Breton, N.; Martinho, M.; Mileo, E.; Belle, V. SimLabel: a graphical user interface to simulate continuous wave EPR spectra from site-directed spin labeling experiments. *Magn. Reson. Chem.* **2017**, *55*, 714-719.

(9) See also: Fantasia, S.; Nolan, S. P. A general synthetic route to mixed NHC-phosphane palladium(0) complexes (NHC = N-heterocyclic carbene). *Chem. Eur. J.* **2008**, *14*, 6987-6993.

(10) Tatsuno, Y.; Yoshida, T.; Otsuka, S. (η^3-Allyl)palladium(II) complexes. *Inorg. Synth.* **1979**, *19*, 220-223.

(11) Navarro, O.; Nolan, S. P. Large-scale one-pot synthesis of N-heterocyclic carbene-Pd(allyl)Cl complexes. *Synthesis* **2006**, 366-367.

(12) McGuinness, D. S.; Cavell, K. J.; Skelton, B. W.; White, A. H. Zerovalent Palladium and Nickel Complexes of Heterocyclic Carbenes: Oxidative Addition of Organic Halides, Carbon−Carbon Coupling Processes, and the Heck Reaction. *Organometallics* **1999**, *18*, 1596-1605.

(13) Morton, J. R.; Preston, K. F. Atomic parameters for paramagnetic resonance data. *J. Magn. Reson.* **1978**, *30*, 577-582.

(14) Koh, A. K.; Miller, D. J. Hyperfine coupling constants and atomic parameters for electron paramagnetic resonance data. *At. Data Nucl. Data Tables* **1985**, *33*, 235-253.

(15) Elgrishi, N.; Rountree, K. J.; McCarthy, B. D.; Rountree, E. S.; Eisenhart, T. T.; Dempsey, J. L. A Practical Beginner's Guide to Cyclic Voltammetry. *J. Chem. Educ.* **2018**, *95*, 197-206.

(16) *iNMR for Mac*, version 6.0; Nucleomatica: Molfetta, Italy, 2016, http://www.inmr.net/ (accessed 11/18/2023).

(17) Solomon, I. Relaxation Processes in a System of Two Spins. *Phys. Rev.* **1955**, *99*, 559-565.

(18) Ming, L. J.; Jang, H. G.; Que, L. 2D NMR studies of paramagnetic diiron complexes. *Inorg. Chem.* **1992**, *31*, 359-364.

(19) Brink, J. M.; Rose, R. A.; Holz, R. C. Characterization of the Structural and Electronic Properties of Spin-Coupled Dinuclear Copper(II) Centers by Proton NMR Spectroscopy. *Inorg. Chem.* **1996**, *35*, 2878-2885.

(20) Evans, D. F. The determination of the paramagnetic susceptibility of substances in solution by nuclear magnetic resonance. *J. Chem. Soc.* **1959**, 2003-2005.

(21) Powers, T. M. In *JoVE Science Education Database. Inorganic Chemistry*; JoVE: Cambridge, MA, 2023.

(22) Sur, S. K. Measurement of magnetic susceptibility and magnetic moment of paramagnetic molecules in solution by high-field fourier transform NMR spectroscopy. *J. Magn. Reson.* **1989**, *82*, 169-173.

(23) Schubert, E. M. Utilizing the Evans method with a superconducting NMR spectrometer in the undergraduate laboratory. *J. Chem. Educ.* **1992**, *69*, 62.

(24) Hoffman, R. E. Variations on the chemical shift of TMS. *J. Magn. Reson.* **2003**, *163*, 325-331.

(25) Bain, G. A.; Berry, J. F. Diamagnetic Corrections and Pascal's Constants. *J. Chem. Educ.* **2008**, *85*, 532-536.

(26) Arentsen, K.; Caddick, S.; Cloke, F. G. N.; Herring, A. P.; Hitchcock, P. B. Suzuki–Miyaura cross-coupling of aryl and alkyl halides using palladium/imidazolium salt protocols. *Tetrahedron Lett.* **2004**, *45*, 3511-3515.

(27) Hruszkewycz, D. P.; Wu, J.; Hazari, N.; Incarvito, C. D. Palladium(I)-Bridging Allyl Dimers for the Catalytic Functionalization of CO_2. *J. Am. Chem. Soc.* **2011**, *133*, 3280-3283.

(28) Sheldrick, G. M. A short history of SHELX. *Acta Crystallogr. Sect. A: Found. Crystallogr.* **2008**, *64*, 112-122.

(29) Farrugia, L. J. WinGX suite for small-molecule single-crystal crystallography. *J. Appl. Crystallogr.* **1999**, *32*, 837-838.

(30) Frisch, M. J.; Trucks, G. W.; Schlegel, H. B.; Scuseria, G. E.; Robb, M. A.; Cheeseman, J. R.; Scalmani, G.; Barone, V.; Mennucci, B.; Petersson, G. A.; Nakatsuji, H.; Caricato, M.; Li, X.; Hratchian, H. P.; Izmaylov, A. F.; Bloino, J.; Zheng, G.; Sonnenberg, J. L.; Hada, M.; Ehara, M.; Toyota, K.; Fukuda, R.; Hasegawa, J.; Ishida, M.; Nakajima, T.; Honda, Y.; Kitao, O.; Nakai, H.; Vreven, T.; J. A. Montgomery, J.; Peralta, J. E.; Ogliaro, F.; Bearpark, M.; Heyd, J. J.; Brothers, E.; Kudin, K. N.; Staroverov, V. N.; Keith, T.; Kobayashi, R.; Normand, J.; Raghavachari, K.; Rendell, A.; Burant, J. C.; Iyengar, S. S.; Tomasi, J.; Cossi, M.; Rega, N.; Millam, J. M.; Klene, M.; Knox, J. E.; Cross, J. B.; Bakken, V.; Adamo, C.; Jaramillo, J.; Gomperts, R.; Stratmann, R. E.; Yazyev, O.; Austin, A. J.; Cammi, R.; Pomelli, C.;

Ochterski, J. W.; Martin, R. L.; Morokuma, K.; Zakrzewski, V. G.; Voth, G. A.; Salvador, P.; Dannenberg, J. J.; Dapprich, S.; Daniels, A. D.; Farkas, O.; Foresman, J. B.; Ortiz, J. V.; Cioslowski, J.; Fox, D. J. *Gaussian 09*, version Revision C.01; Gaussian, Inc.: Wallingford CT, 2010.

(31) Becke, A. D. A new mixing of Hartree–Fock and local density-functional theories. *J. Chem. Phys.* **1993**, *98*, 1372-1377.

(32) Becke, A. D. Density-functional thermochemistry. III. The role of exact exchange. *J. Chem. Phys.* **1993**, *98*, 5648-5652.

(33) Hay, P. J.; Wadt, W. R. Ab initio effective core potentials for molecular calculations. Potentials for the transition metal atoms Sc to Hg. *J. Chem. Phys.* **1985**, *82*, 270-283.

(34) Wadt, W. R.; Hay, P. J. Ab initio effective core potentials for molecular calculations. Potentials for main group elements Na to Bi. *J. Chem. Phys.* **1985**, *82*, 284-298.

(35) Hay, P. J.; Wadt, W. R. Ab initio effective core potentials for molecular calculations. Potentials for K to Au including the outermost core orbitals. *J. Chem. Phys.* **1985**, *82*, 299-310.
